# Native top-down mass spectrometry provides insights into the copper centers of membrane-bound methane monooxygenase

**DOI:** 10.1038/s41467-019-10590-6

**Published:** 2019-06-17

**Authors:** Soo Y. Ro, Luis F. Schachner, Christopher W. Koo, Rahul Purohit, Jonathan P. Remis, Grace E. Kenney, Brandon W. Liauw, Paul M. Thomas, Steven M. Patrie, Neil L. Kelleher, Amy C. Rosenzweig

**Affiliations:** Departments of Molecular Biosciences and of Chemistry, Northwestern University, 2205 Tech Drive, Evanston, IL 60208 USA

**Keywords:** Metalloproteins, Membrane proteins, Mass spectrometry

## Abstract

Aerobic methane oxidation is catalyzed by particulate methane monooxygenase (pMMO), a copper-dependent, membrane metalloenzyme composed of subunits PmoA, PmoB, and PmoC. Characterization of the copper active site has been limited by challenges in spectroscopic analysis stemming from the presence of multiple copper binding sites, effects of detergent solubilization on activity and crystal structures, and the lack of a heterologous expression system. Here we utilize nanodiscs coupled with native top-down mass spectrometry (nTDMS) to determine the copper stoichiometry in each pMMO subunit and to detect post-translational modifications (PTMs). These results indicate the presence of a mononuclear copper center in both PmoB and PmoC. pMMO-nanodisc complexes with a higher stoichiometry of copper-bound PmoC exhibit increased activity, suggesting that the PmoC copper site plays a role in methane oxidation activity. These results provide key insights into the pMMO copper centers and demonstrate the ability of nTDMS to characterize complex membrane-bound metalloenzymes.

## Abstract (web summary)

The activity of the membrane-bound enzyme pMMO depends on copper but the location of the copper centers is still under debate. Here, the authors reconstitute pMMO in nanodiscs and use native top-down MS to localize its copper centers, providing insights into which sites are essential for activity.

## Introduction

Particulate methane monooxygenase (pMMO) is an integral membrane metalloenzyme that oxidizes methane to methanol^1^ in methanotrophic bacteria^2^. pMMO comprises three subunits, PmoB, PmoA, and PmoC, assembled into a larger α_3_β_3_γ_3_ complex^3–7^. The enzymatic activity of pMMO depends on the presence of copper, with approximately two copper ions per αβγ protomer required for optimal activity^3,8^. Extensive efforts have been devoted to elucidating the nature of the pMMO copper active site^1,9^, with the ultimate goal of identifying the reactive copper-oxygen intermediate responsible for activating the 105 kcal/mol C–H bond in methane^1^. A molecular and mechanistic understanding of the pMMO active site is essential for the design of methane remediation tools, including synthetic catalysts and engineered methanotrophs^10,11^, and may also provide insight into copper-mediated oxidation chemistry.

**Fig. 1:**
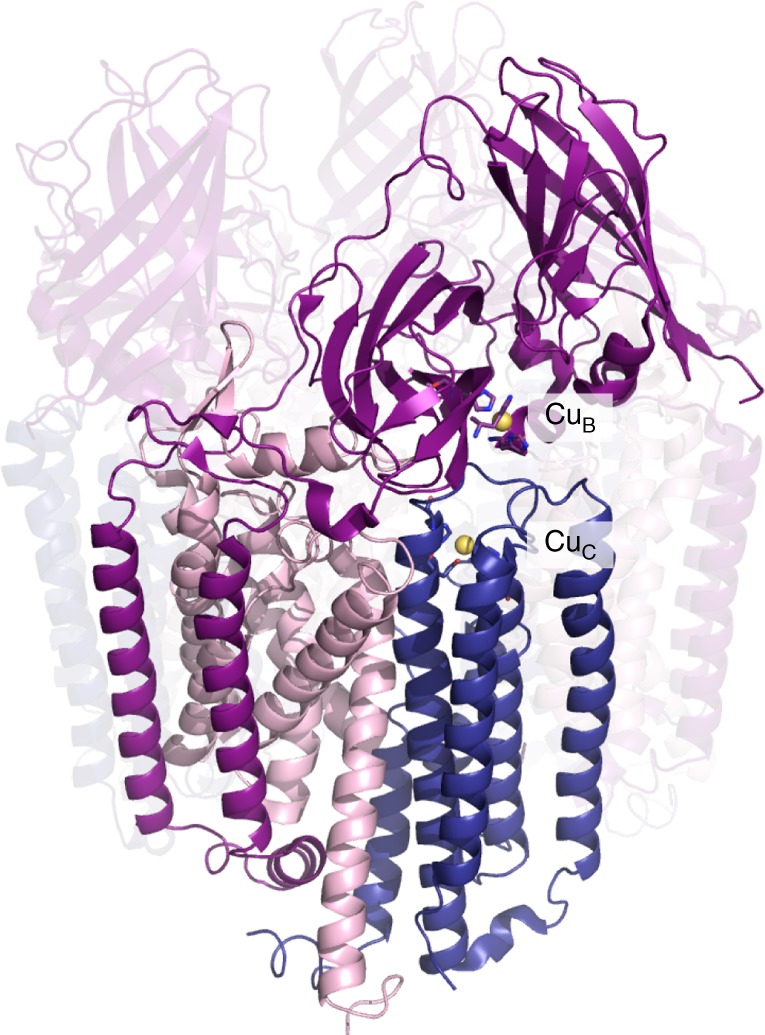
The pMMO structure and location of the metal centers. In the crystal structure of *Methylocystis* sp. strain Rockwell-pMMO (4PHZ), there is a monocopper center in PmoB coordinated by residues His 29, His 133, and His 135 (Cu_B_), and a monocopper center in PmoC coordinated by Asp 129, His 133, and His 146 (Cu_C_). PmoA, PmoB, and PmoC are shown in pink, purple, and blue, respectively, with one of the three protomers highlighted. Copper ions are shown as yellow spheres

Candidate locations for the copper active site were first identified in the crystal structure of *Methylococcus capsulatus* (Bath) pMMO (Bath-pMMO), which revealed three metal centers^6^. Two copper centers were modeled in PmoB: a nonconserved monocopper site ligated by His 48 and His 72 (bis-His site) that is not observed in other pMMO structures, and a conserved site at the amino terminus ligated by His 33, His 137, and His 139 (Cu_B_ site). The latter site was initially modeled as dicopper on the basis of extended X-ray absorption fine structure (EXAFS) data^3,5,12^, but later analysis and crystal structures indicated that this site may instead be monocopper (Fig. 1)^3,4,13^. In addition, a third site occupied by zinc from the crystallization buffer was present in the PmoC subunit with ligands Asp 127, His 131, and His 144. This site, sometimes called the variable metal binding site, can also be occupied by copper^3^ and is located in a chronically disordered region of the PmoC subunit^4^.

The nuclearity of the Cu_B_ site has been defined unambiguously by recent in vivo advanced electron paramagnetic resonance (EPR) spectroscopic characterization of *Methylococcus capsulatus* (Bath). In addition, the presence of a second monocopper center at the PmoC variable metal binding site, denoted the Cu_C_ site, was demonstrated using double electron-electron resonance (DEER) spectroscopy^14^. These data established an important correlation between the sites observed in the crystal structure and the sites present in the cell. While the Cu_B_ site was previously assigned as the active site^8^, our more recent studies indicate that it is not sufficient for methane oxidation^14^, consistent with the requirement for two copper ions^3,8^. The possibility that methane oxidation occurs at the PmoC Cu_C_ site has been raised^14^, but lacks direct experimental support.

The study of pMMO has been hindered by the limitations of traditional biochemical, structural, and spectroscopic approaches. Metal analyses indicating the presence of 2–3 copper ions provide no insight into the specific locations of these metal ions, necessitating inferences based on combined crystallography and spectroscopy. The crystal structures are subject to artifacts from the crystallization buffer, such as the presence of zinc in the PmoC site^5,6^ as well as unknown effects of detergent solubilization and the crystallization process. In addition, some flexible regions are never observed in the electron density maps^4^. Spectroscopic data collected on pMMO reflect a mixture of copper species, rendering it nontrivial and in the case of EXAFS, impossible, to separate signals arising from different sites. This issue is compounded by the fact that pMMO has not been expressed heterologously, precluding facile site-directed mutagenesis. These challenges are not specific to pMMO; determination of metal stoichiometry and localization can be a major challenge for large, multisubunit metalloprotein complexes.

**Fig. 2:**
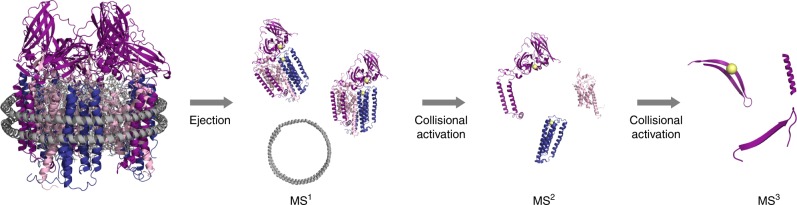
The nTDMS platform for pMMO characterization. The pMMO-nanodisc complex is subjected to ejection from the nanodisc using collision-induced dissociation (CID) at the source resulting in the stabilization of the pMMO protomer species (MS^1^). Increasing collisional activation breaks up the protomer into individual pMMO subunits (MS^2^). Further collisional activation enables backbone fragmentation of each subunit using higher energy collisional dissociation (HCD) in the HCD cell of the instrument (MS^3^)

An emerging alternative approach for metal center characterization is native mass spectrometry (nMS), which typically employs electrospray ionization (ESI) at neutral pH from volatile, non-reducing buffers^15^, and instrument settings that faithfully preserve the primary and quaternary composition of complexes in the sample^16,17^. Coupling tandem MS (MS^n^) activation of a non-covalent protein assembly to the nMS analysis^18^ can help to characterize liberated components from the complex, such as subunits^18^. Moreover, measurement of intact mass values by nMS followed by gas-phase protein fragmentation, termed native top-down mass spectrometry (nTDMS)^19,20^, enables the identification and characterization of specific proteoforms emanating from encoding genes including those containing underlying sequence changes due to polymorphisms, unexpected truncations, or post-translational modification (PTM)^19–24^. In particular, nTDMS can be used to determine metal stoichiometry of each subunit and even the identities of the metal binding ligands^25–27^. In 2013^19^, a nTDMS platform achieved a three-tiered tandem mass spectrometry (MS) process, comprising measurement of an intact protein complex (MS^1^), analysis of ejected monomer(s) (MS^2^), and backbone fragmentation of each monomer (MS^3^, or pseudo-MS^3^) measured at isotopic resolution (Fig. 2)^19,20^.

Here we apply nTDMS to pMMO. While some membrane proteins have been characterized by nMS^28–31^, this study involves nTDMS characterization of a multisubunit, membrane metalloenzyme via MS^3^ fragmentation of individual subunits for metal ion identification, localization, and stoichiometric determination. Our nTDMS analysis of pMMO in micelles and nanodiscs^32^ identifies proteoforms of pMMO subunits and PTMs that may have functional implications. Most importantly, the data support the presence of one copper ion each in the PmoB and PmoC subunits, and in combination with activity profiles of pMMO in nanodiscs, suggest that the copper ion bound to PmoC is important for the oxidation of methane to methanol.

## Results

### Defining the proteoform composition of pMMO by nTDMS

Our initial nTDMS studies focused on pMMO from *Methylomicrobium alcaliphilum* 20Z (20Z-pMMO). Methanotrophs from the genus *Methylomicrobium (Mm.)* have attracted interest as tractable model systems in engineering applications for methane-to-biofuel conversion, and *Mm. alcaliphilum* 20Z has been the subject of several studies^33–35^, including the recent characterization of its pMMO^4^. In the crystal structure of 20Z-pMMO, one copper ion was modeled into the Cu_B_ site, supported by EXAFS analysis. However, metal quantitation and EPR analysis indicated the presence of two copper ions per pMMO protomer^4^. In the crystal structure, the PmoB bis-His site is unoccupied and the PmoC subunit is mostly disordered, rendering it impossible to determine if it houses any metal ions. Thus, additional data are needed to assess the metal centers in 20Z-pMMO, motivating its investigation by nTDMS.

**Fig. 3:**
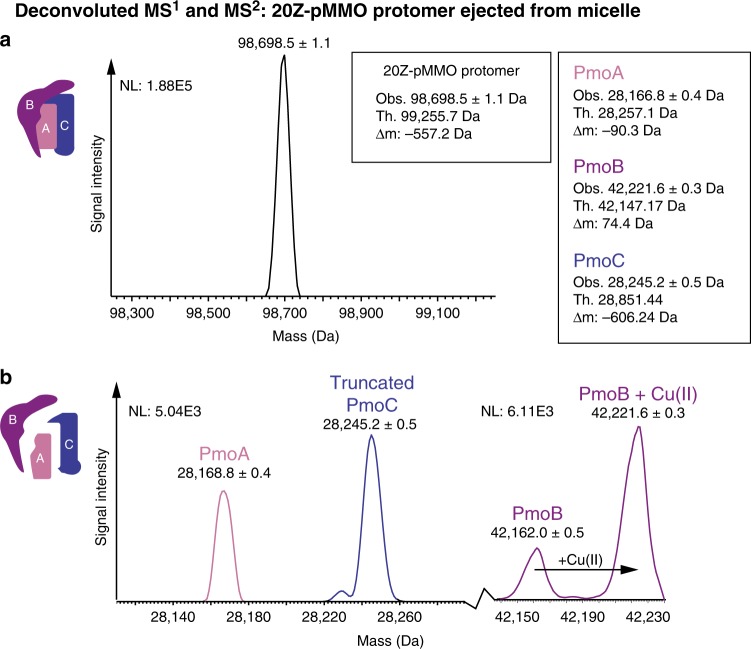
nTDMS analysis of 20Z-pMMO in Triton X-100 micelles. **a** Deconvoluted MS^1^ of 20Z-pMMO protomer upon ejection from a Triton X-100 micelle. Charge state deconvolution of the major species yields a mass of 98,696.5 ± 1.1 Da. The theoretical mass is derived from the unmodified subunits of pMMO and accounts for the cleavage of a known signal peptide in PmoB. **b** Deconvoluted MS^2^ of 20Z-pMMO subunits ejected from the 16+ charge state of the protomer after activation by collisions with neutral gas. The spectrum shows detection of three species, labeled pink, purple, and blue and assigned to PmoA, PmoB, and PmoC, respectively. Adding the measured masses of the ejected subunits yields 98,633.6 Da, which is 62.4 Da smaller than the major protomer mass measured in the MS^1^. NL values reflect maximum signal intensity in the spectrum

20Z-pMMO was solubilized from as-isolated membranes and purified by anion exchange chromatography^4^. After reconstituting 20Z-pMMO in Triton X-100 micelles, the complex was subjected to nMS analysis for characterization. The MS^1^ analysis (Supplementary Fig. 1a) produced a species exhibiting a 15–18+ charge state distribution. Deconvolution of the charge states of the major species present at 93% (Supplementary Fig. 1a, labeled in purple) yielded a mass of 98,696.0 ± 1.1 Da (± represents standard deviation from the reported average mass calculated by sampling multiple charge states of the protein species) (Fig. 3a). The theoretical mass of an unmodified pMMO protomer, 99,255.7 Da, is based on the amino acid sequences of the subunits, assuming the cleavage of a known signal peptide at the PmoB N-terminus^36^ (theoretical mass of a protomer with an uncleaved PmoB is 102,638.7 Da). While the observed mass of the predominant species is lower than the theoretical mass that assumes cleavage of the PmoB signal peptide (mass difference, Δm *=* −559.7 Da), the close match suggests that the species ejected from the Triton X-100 micelle is a modified pMMO protomer.

To investigate the possible presence of PTMs and metal cofactors in proteoforms comprising the pMMO protomer, the 16+ charge state of the pMMO-micelle complex was subjected to nTDMS. First, intact pMMO protomer was activated by collisions with neutral gas in the ESI source region to produce a pseudo-MS^2^ of the ejected subunits (Supplementary Fig. 1b). Three major protein species that correspond closely in mass to each one of the pMMO subunits were detected (Fig. 3b). The PmoA subunit, with a theoretical mass of 28,257.1 Da, was tentatively assigned to the 28,166.8 ± 0.4 Da proteoform, with a Δm of −89.1 Da consistent within a dalton for the removal of the initiator methionine (Met_OFF_) and the addition of N-terminal acetylation (NtAc) to the new N-terminus. The 42,221.6 ± 0.3 Da proteoform, which is closest in mass to PmoB (theoretical mass 42,147.17 Da, Δm =  + 74.4 Da), could result from the replacement of two protons by one copper(II) ion^4^ (+61.5 Da) and a potential PTM. The third species had a mass of 28,245.2 ± 0.5 Da, which may be attributable to a truncated proteoform of PmoC (theoretical mass 28,851.4 Da, Δm *=* −606.2 Da). The addition of the three detected masses yields an αβγ protomer of 98,633.6 Da, which is 62.4 Da smaller than the observed protomer via MS^1^. This mass loss suggests that a copper ion (theoretical average mass of 61.5 Da) may be lost upon subunit ejection from the pMMO-micelle complex.

To confirm subunit assignments and characterize their mass shifts, intact proteoforms need to be fragmented by tandem MS. Attempts at tandem MS on subunits ejected from the Triton X-100 micelle were unsuccessful due to the limited ability to activate ions in the ESI source; for example, disrupting the micelle to produce intact pMMO ions required relatively high settings of 150–195 V. This precluded further activation of the complex in the ESI source (200 V max), which is essential for individual subunits to be isolated and further dissociated in the higher energy collision dissociation cell (HCD) within the instrument.

### nTDMS analysis of pMMO proteoforms ejected from nanodiscs

**Fig. 4:**
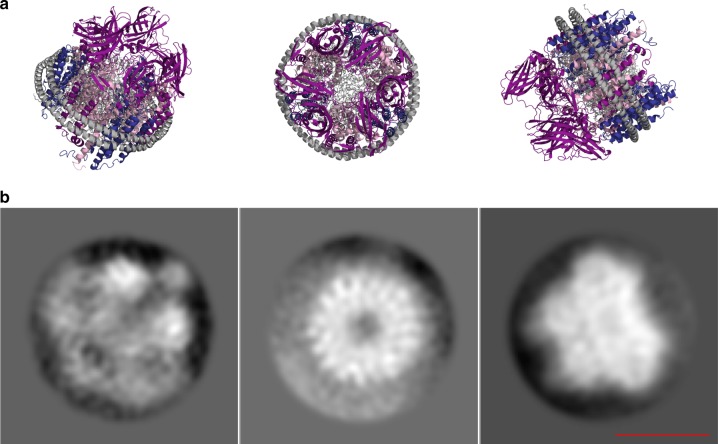
Cryo-EM 2D class averages of 20Z-pMMO in MSP2N2 nanodiscs. **a** A model of a pMMO nanodisc complex generated from the crystal structures of *Methyloccocus capsulatus* (Bath) pMMO (PDB accession code 3RGB) and MSP1D1 (from PDB accession code 6CC9). This model is for illustrative purposes and is not from 2D class averages. **b** Tilted, top, and side views of 2D class averages of 20Z-pMMO reconstituted in nanodiscs. A representative scale bar is shown as a 10 nm red line

To achieve further collisional activation and to potentially stabilize the copper ion lost upon subunit ejection, Triton X-100 micelles were substituted with nanodiscs, discoidal lipid bilayers absent of detergent and commonly used to stabilize membrane proteins^37^. Nanodiscs have been reported to minimize coulomb-induced unfolding in the gas phase, suggesting that they can be protective of labile non-covalent interactions upon ejection from the assembly^29–31^. 20Z-pMMO was embedded in nanodiscs using POPC lipids and the membrane scaffold proteins MSP2N2 or MSP1E3D1 (Supplementary Figs 2–3)^32^. Nanodiscs formed using MSP2N2 and MSP1E3D1 have diameter distances of up to 17 and 12 nm, respectively, and can accommodate the 9 nm pMMO complex. MSP1E3D1 provided higher reconstitution yields and stability than MSP2N2, potentially because the smaller diameter of MSP1E3D1 allows for a tighter fit with pMMO. Two-dimensional class averages of cryo-electron microscopy images (cryo-EM) and SDS-PAGE were used to assess reconstitution and confirm the presence of all three pMMO subunits in MSP2N2 nanodiscs (Fig. 4, Supplementary Fig. 4); MSP1E3D1 nanodiscs were analyzed by negative stain electron microscopy images and SDS-PAGE.

**Fig. 5:**
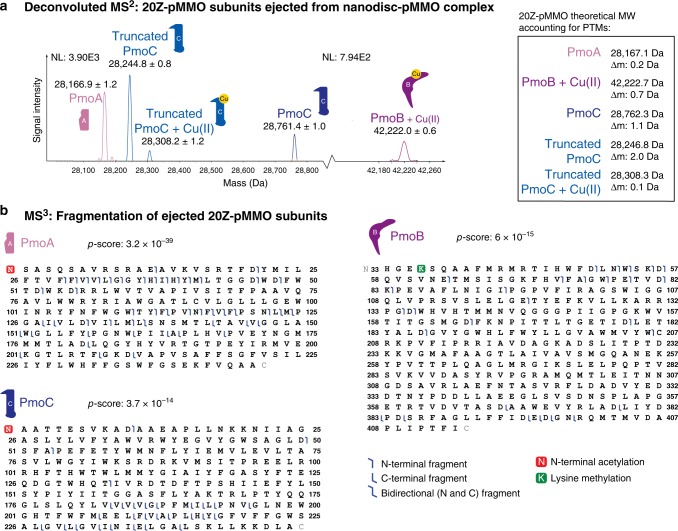
nTDMS analysis of 20Z-pMMO in MSP2N2 nanodiscs. **a** Deconvoluted MS^2^ showing detection of 20Z-pMMO subunit masses upon ejection from the nanodisc-pMMO complex. The panel on the right contains the theoretical masses of the subunits, accounting for the modifications characterized by tandem MS. **b** Graphical fragment maps of pMMO subunits derived from the MS^3^ step in the nTDMS platform. Fragments are depicted as blue flags in the graphical fragment maps, indicating which regions of the protein sequence can be accounted for in mass by the fragments. The pMMO subunits were found to be modified by N-terminal acetylation (PmoA and PmoC), N-terminal truncation (PmoC), and lysine methylation (PmoB). The graphical fragment map of PmoB begins at residue His 33, showing cleavage of the known N-terminal signal peptide

The 20Z-pMMO nanodisc complex was subjected to the nTDMS platform, which presented some initial challenges. First, we were unable to obtain an intact mass of the complex given the heterogeneity of the signals detected. Second, excess of lipids in the nanodiscs led to the detection of lipid clusters, which dominated the signal in MS^1^ spectra. We overcame these difficulties by titrating the lipid content in the nanodiscs to an optimal concentration that reduced signals from lipid clusters, as measured by nMS, while still preserving enzymatic activity. Additionally, we tuned the ion optics of the mass spectrometer, including the C-trap entrance lens voltage, to filter out high intensity lipid cluster ions. The pMMO complex was subjected to subunit ejection from the nanodisc complex using collision-induced dissociation (CID) at the source. The successful ejection of the individual subunits (Supplementary Fig. 5) with 195 eV of source fragmentation enabled their subsequent isolation by the quadrupole mass filter and fragmentation in the HCD cell. A deconvoluted MS^2^ spectrum shows detection of protein species that were targeted for fragmentation (Fig. 5a). HCD generated *b* and *y* ions from fragmentation at backbone amide positions that were mapped onto the sequence of the pMMO subunits (Fig. 5b), thereby localizing mass shifts^38^.

Notably, as shown in the graphical fragment maps in Fig. 5b, PmoB includes residues His 33 to Ile 414, consistent with the presence of a leader sequence that is cleaved post-translationally^36^. PmoB residue Lys 36 was found to be methylated, a modification localized by the nTDMS to residues 33–51 and pinpointed by tandem MS of pepsin-digested peptides of 20Z-pMMO (Supplementary Fig. 6a). This lysine is located 8.3 Å from the Cu_B_ site and does not appear methylated in the electron density map^4^, yet was present at ~100% stoichiometry from the nTDMS data. It is not known whether this methylation is functionally important. PmoA was characterized to be Met_OFF_ and NtAc (Fig. 5b), as suggested by analysis of the micelle sample. The MS^2^ from the nanodisc reflects two populations of PmoC. A minor species is observed with Met_OFF_ and NtAc while the major PmoC species is a truncated form without the first six N-terminal residues (MAATTE) (Fig. 5a, Supplementary Fig. 6b).

### Localization of the copper-binding sites in pMMO

We next sought to determine the stoichiometry of metal binding to each pMMO subunit. The copper in 20Z-pMMO was determined by EPR analysis to be predominantly in the Cu(II) oxidation state^4^. Deconvolution of the MS^2^ generated upon ejection of pMMO from the nanodisc (Fig. 5a) revealed that the predominant PmoB proteoform has a mass consistent with a methylation (14 Da) and one Cu(II) ion (61.5 Da). Given that one copper ion remains associated with PmoB even after subunit ejection, we hypothesized that some copper might remain bound to fragments generated in the pseudo-MS^3^ analysis^19^ of this proteoform, thereby helping to verify the location of the Cu_B_ site^27^. Two *b*-type fragment ions, *b*_165_ and *b*_186_ (corresponding to the numbering indicated in the graphical fragment map in Fig. 5b), were identified with mass shifts consistent with the binding of one Cu(II) ion (Supplementary Fig. 7) as demonstrated by the fitting of the theoretical isotopic distributions for copper-bound fragment ions. Notably, both copper-bound fragment ions occurred C-terminal to an aspartic acid residue, which is consistent with known fragmentation propensities under native ESI^22^. No copper binding was observed for fragment *b*_135_ or for any other downstream *b* ions. In congruence with the crystal structure, these copper-bound fragment ions suggest that the copper-binding region (green underline in Supplementary Fig. 7) may be confined to the region spanning Trp 136-Asp 186, which contains the coordinating residues His 137 and His 139, but not the ligands to the bis-His site, His 48 and His 72. Copper binding fragment ions containing His 33 were not observed, perhaps due to the labile nature of this ligand suggested by the crystal structures^3–6^. Moreover, there is no copper binding observed in the C-terminal cupredoxin domain, previously suggested to bind ~10 copper ions^39^.

PmoC ejected from the pMMO-nanodisc complex has a proteoform present at 16% relative abundance that is consistent with a copper ion bound to the truncated PmoC species (Fig. 5a). Reduced copper stoichiometry in pMMO is observed upon nanodisc reconstitution (Supplementary Fig. 8), suggesting that bound copper may be lost from PmoC during the reconstitution. To determine whether the copper-binding stoichiometry of PmoC could be increased by exogenous addition of copper to the electrospray buffer, we added 1, 3, and 6 molar equivalents (eq.) of Cu(II) per protomer to 20Z-pMMO in nanodiscs and analyzed these samples by nTDMS (Supplementary Fig. 9). We found that at 1 eq. of Cu(II) per protomer, 10.4% (±3%) of PmoC is bound to copper. At 3 eq. of Cu(II), copper binding increases to a maximum of 27% (±3%), with no further increase after addition of more copper. Notably, no additional copper binding is observed for PmoB, as shown in the inset of Supplementary Fig. 9b. The precursor intensity of copper-bound PmoC proteoform was too low for these samples and thus was not fragmented successfully for metal localization. Taken together, the spectra generated from the micelle and nanodisc systems indicate that 20Z-pMMO binds one copper ion near the PmoB N-terminus and one copper ion in the PmoC subunit.

To validate the copper localization in 20Z-pMMO, pMMOs from other methanotrophs were investigated via nTDMS. Interestingly, PmoC copper binding was observed for samples of pMMO from *Mm. buryatense* 5GB1C (5G-pMMO) in Triton X-100 micelles. The MS^1^ analysis of 5G-pMMO shows a predominant mass species that correlates to a pMMO protomer bound to two copper ions (Supplementary Figs. 10, 11a), similar to 20Z-pMMO in micelles. MS^2^ ejection (Supplementary Figs. 11b, 12) of the subunits confirms that the predominant species of PmoB also contains one copper ion and has Lys 36 methylation. While MS^2^ of 5G-pMMO in micelles indicated copper binding to the PmoC subunit, this was not the case for the 20Z-pMMO PmoC in micelles, possibly due to structural differences between the two PmoC subunits. Unfortunately, 5G-pMMO in nanodiscs exhibited poor ejection from the nanodisc complex and could not be characterized via nTDMS.

**Fig. 6:**
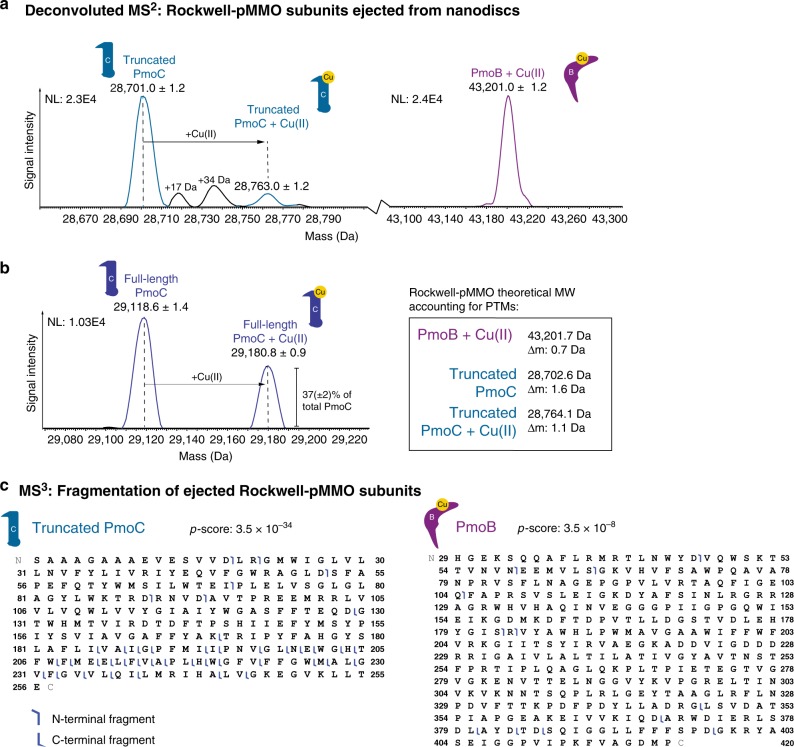
nTDMS analysis of Rockwell-pMMO in MSP1E3D1 nanodiscs. **a** Deconvoluted MS^2^ demonstrating detection of Rockwell-pMMO subunit masses upon ejection from the nanodisc without or **b** with copper supplementation during nanodisc reconstitution. The panel on the right contains the theoretical masses of the subunits, accounting for the modifications characterized by tandem MS. The two species shifted in mass from PmoC by + 17 and + 34 Da likely correspond to the replacement of one and two protons by ammonium adducts (Th. 17.03 Da and 34.06 Da, respectively) on PmoC commonly observed in nESI^65^. **c** The MS^3^ of truncated PmoC and PmoB yielded fragment ions, depicted as blue flags in the graphical fragment maps that indicate which regions of the protein sequence can be accounted for in mass by the fragments. Truncated PmoC lacks the first five residues of the N-terminus (MSSTT), and its graphical fragment map begins at residue Ser 6. The graphical fragment map of PmoB begins at residue His 29, consistent with the cleavage of the known N-terminal signal peptide

We then investigated pMMO from *Methylocystis* sp. strain Rockwell (Rockwell-pMMO). In the crystal structure of Rockwell-pMMO, the PmoC site is occupied by copper^3^. Unlike 5G-pMMO, Rockwell-pMMO in MSP1E3D1 nanodiscs (Supplementary Fig. 13) ejected well from the nanodisc complex and was thus amenable to nTDMS analysis of the subunits. Subunit ejection (Supplementary Fig. 14, Fig. 6a) and subsequent fragmentation of Rockwell-pMMO led to the identification of the PmoB and PmoC subunits (Fig. 6c). Similar to 20Z-pMMO, PmoB showed an intact mass shift of 61.5 Da that suggests 100% occupancy of a single Cu(II) ion. Unlike 20Z- and 5G-pMMO, PmoB methylation was not detected, indicating that this PTM may not be necessary for activity and may be specific to *Methylomicrobium* pMMOs. Truncated PmoC was observed in the apo form, but close examination of the spectrum (Fig. 6a) reveals a low abundance peak that is shifted by the mass of copper, consistent with inductively coupled plasma mass spectrometry (ICP-MS) data (vide infra) and with a labile metal interaction that is partially lost at high collision voltages or during nanodisc reconstitution, as observed for 20Z-pMMO.

### Linking pMMO activity to copper binding by PmoC

**Fig. 7:**
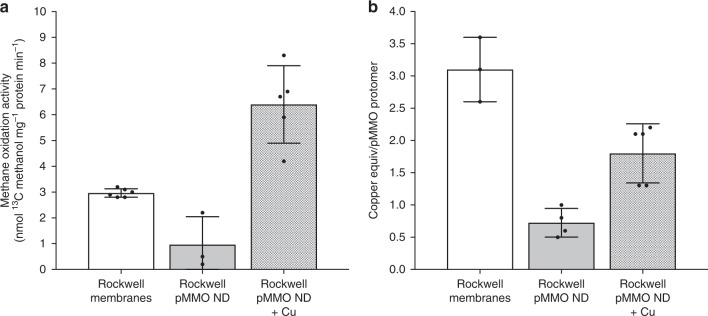
Activity and metal content of Rockwell-pMMO in nanodiscs. **a** ^13^C methane oxidation activity of Rockwell-pMMO in membranes and in nanodiscs (ND) without or with copper supplementation. Values are shown in nmol ^13^C methanol•mg^−1^ protein•min^−1^. Error bars represent standard deviation of individual measurements (black dots) of *n* = 6, 3, and 5, respectively. **b** Copper equivalents per pMMO protomer for Rockwell-pMMO in membranes and in nanodiscs without or with copper supplementation. Error bars represent standard deviation of individual measurements (black dots) of *n* = 3, 4, and 5, respectively. Source data are provided as a Source Data file

In the crystal structures, the PmoC metal binding site is occupied by zinc^3,5–7^ or copper (Rockwell-pMMO)^3^, or is completely disordered^4^. However, it has thus far been unclear whether metal binding at this site is functionally relevant^3^. Given that the nTDMS data indicate the presence of some copper in the PmoC subunits from three different organisms, and that pMMO activity requires more than one copper ion, we investigated the effect of exogenous copper addition on Rockwell-pMMO by both nTDMS and activity assays. Membrane-bound Rockwell-pMMO exhibits the highest methane oxidation activity at 30 °C out of all characterized pMMOs^3,4^, but upon reconstitution into nanodiscs, the activity dramatically decreased compared to that of the as-isolated membranes (Fig. 7a).

Upon addition of one equivalent of CuSO_4_ per pMMO protomer during the nanodisc reconstitution process, the methane oxidation activity of the pMMO-nanodisc complex increased six-fold (Fig. 7a). The activity was higher than that of membrane-bound pMMO using duroquinol as a reductant, similar to the increased activity observed for pMMOs reconstituted into bicelles^4^. ICP-MS analysis of the pMMO-nanodisc samples prepared with and without copper supplementation showed that in the absence of copper supplementation, 0.7 ± 0.2 (*n* = 4) copper ion per pMMO protomer is present (Fig. 7b). Upon copper addition and incorporation into the pMMO-nanodisc complex, the copper content increased to 1.8 ± 0.5 (*n* = 5) copper ions per pMMO protomer, consistent with the presence of up to two copper-binding sites. These copper-supplemented Rockwell-pMMO nanodisc samples were then analyzed by nTDMS to determine the location of the second copper ion (Fig. 6b, Supplementary Fig. 15). The nTDMS analysis of the active Rockwell-pMMO prepared with exogenous addition of copper showed a significantly higher amount of copper bound PmoC (37 ± 2%, Fig. 6a) compared to the inactive sample (Fig. 6a). The correlation between this result and the activity data suggests that the copper ion in PmoC, likely corresponding to the spectroscopically assigned Cu_C_ site^14^, is essential for methane oxidation activity.

## Discussion

The nTDMS analysis presented here advances our understanding of pMMO in several ways. First, we have obtained accurate intact masses for the pMMO protomer and individual subunits and have detected a previously unknown methylation of residue Lys 36 in the PmoB subunits of 20Z-pMMO and 5G-pMMO. The importance of this PTM, which is not found in Rockwell-pMMO, is unclear. This methylation might afford protection against reaction with radicals and oxidative damage, as proposed for methyl-coenzyme M reductase^40^ and lytic polysaccharide monooxygenases^41^, or provide additional hydrophobic interactions important for methane binding. Alternatively, this methylated lysine may be an off-target product formed by methyltransferases responsible for synthesizing osmolytes in haloalkaliphiles^42^. The observation of these modifications, including the truncated proteoforms of PmoC, highlights the importance of measuring the intact masses of proteoforms, as such modifications were not readily observed in previous biochemical studies^4^ and may have functional implications.

Second, we have localized the copper sites in pMMO and have established a correlation between the PmoC site and methane oxidation activity. The observation of one copper ion in PmoB and one copper ion in PmoC is consistent with in vivo EPR and DEER data^14^, and confirms that the two copper ions detected in pMMO by metal analysis occupy these two sites. Previous studies have suggested that the PmoC site is important for activity. In particular, mutagenesis of the PmoC metal binding ligands in hydrocarbon monooxygenase (HMO), a homolog of pMMO, abrogates enzyme activity^43^. Additionally, inhibition of pMMO by zinc has been attributed to zinc binding in the PmoC site^3^. However, the different occupancies of this site in the crystal structures, including the presence of zinc^3,5–7^, copper^3^, or no observable metal ion^4^, have obscured its biological relevance. Here we show that copper and activity loss upon nanodisc reconstitution can be restored by addition of exogenous copper, which increases copper occupancy of the PmoC site (Figs 6, 7). These results suggest that the PmoC Cu_C_ site plays an important role in methane oxidation. Disruption of the native membrane may account for the increased lability of this site as compared to the consistently observed Cu_B_ site, which is in the periplasmic region of PmoB.

The Cu_C_ site and the respective roles of both monocopper sites in pMMO must be investigated further. The Cu_C_ site resides in a disordered yet highly conserved region of PmoC, and additional information on its coordination environment is desirable. It may be that the two monocopper centers function analogously to the non-coupled monocopper sites in peptidylglycine α-hydroxylating monooxygenase and dopamine β-monooxygenase^44^, in which the Cu_M_ site is involved in O_2_ activation and the Cu_H_ site, located 11 Å away across a solvent-filled cleft, provides the second electron to the Cu_M_ site for turnover. In the pMMO crystal structures, the PmoB and PmoC metal centers are 20 Å apart, but in the membrane, the PmoB periplasmic domain may shift towards the lipid bilayer, altering this distance. The combined activity and nTDMS data show that increased loading of the Cu_C_ site enhances activity, consistent with a recent proposal that methane and oxygen bind at this site^14^. It remains unclear whether the Cu_B_ site is absolutely necessary for activity. While this site is consistently observed, its three histidine ligands are not present in the verrucomicrobial pMMO PmoB sequences^45^, suggesting that methane oxidation may occur in its absence. In comparison, the Cu_C_ ligands are conserved in all pMMOs^45,46^, and its location in the intracytoplasmic membrane may facilitate methane access^47^.

It is also important to note that the activity of the Rockwell-pMMO nanodisc complex (Fig. 7a) is significantly lower than that of whole cells^1,3^, which could result from loss of additional copper sites prior to nanodisc reconstitution and nTDMS analysis. However, recent spectroscopic analysis indicates that the Cu_B_ and Cu_C_ sites are also present in whole cells^14^, suggesting that the activity loss is primarily due to other factors, such as loss of potential interacting partners^48^, use of a nonphysiological reductant^49^, or disruption of the membrane structure and composition^4,50^. These considerations may differentially affect the two monocopper sites.

Through an alternative approach coupling membrane mimetics with nTDMS analysis, we have gained valuable insights into the active form of pMMO. The nTDMS platform relies on the isolation of distinct mass peaks using a quadrupole mass filter optimized for selection of high *m/z* ions produced by native electrospray^19^. Native-mode TDMS also circumvents protein contamination issues, is sensitive to the dynamic nature of large protein assemblies, and can resolve functionally different proteoforms of metal-bound subunits. The technological advances described here can overcome difficulties intrinsic to membrane proteins^51^ and challenges associated with nanodisc samples^29,31^. Moreover, the ability to localize metal ions fills a gap in the traditional bioinorganic toolbox to characterize metalloproteins. Therefore, the experimental findings not only impact pMMO, but have broad implications for the improved characterization of myriad challenging membrane-bound complexes in metallobiology.

## Methods

### Membrane scaffold protein expression and purification

The membrane scaffold proteins MSP1E3D1 and MSP2N2, each with TEV-cleavable N-terminal 7-histidine tags, were expressed and purified^32^. *E. coli* BL21(DE3) cells were transformed with plasmids pMSP1E3D1 (Addgene, #20066) or pMSP2N2 (Addgene, #29520), and 1 L cultures were inoculated at a starting OD_600_ of 0.1 in Terrific Broth media and grown for 4 h at 37 °C with shaking at 200 rpm until an OD_600_ of 2.3 was reached. The cultures were induced with 1 mM isopropyl ß-D-1-thioglactopyranoside (IPTG) (RPI) and were grown for an additional 4 h at 37 °C for protein expression, yielding 5 g wet cell pellets per L of culture. Cell pellets were harvested by centrifugation at 8000×*g* for 10 min, flash-frozen in liquid nitrogen, and stored at −80 °C.

Cells were resuspended in buffer A (40 mM Tris, pH 7.3, 250 mM NaCl, 20 mM imidazole) at 50 mL per 10 g cell pellet. EDTA-free protease inhibitor cocktail (1 tablet per 50 mL, Roche) and chicken egg lysozyme (10 mg per 50 mL, Sigma-Aldrich) were added. Once resuspended, Triton X-100 (Sigma-Aldrich) was added to a final concentration of 1% v/v and stirred. Cells were lysed on ice by sonication at 35% amplitude for 10 min (1 s on, 1 s off) and cell debris was removed by centrifugation at 10,000×*g* for 30 min. The soluble fraction was loaded onto a column containing 10 mL Ni-NTA beads (Qiagen). The beads were washed with 50 mL of buffer B (40 mM, Tris pH 7.3, 250 mM NaCl, 20 mM imidazole, 50 mM sodium cholate) followed by 150 mL buffer A. The protein was then eluted with buffer C (40 mM Tris, pH 7.3, 250 mM NaCl, 250 mM imidazole).

The elution fractions were pooled and TEV protease was added at a mass ratio w/w of 1:40 TEV:protein. This mixture was dialyzed using a 10 MWCO SnakeSkin dialysis tubing (Thermo Fisher Scientific) in 1 L of buffer D (40 mM Tris, pH 7.3, 250 mM NaCl, 20 mM imidazole, 1 mM EDTA) overnight with a buffer change after 1 h. TEV protease was removed by applying the sample to a Ni-NTA column and collecting the flowthrough, which was then dialyzed overnight against 1 L of buffer E (25 mM PIPES, pH 7.3, 250 mM NaCl) with a buffer change after 1 hr. The purified MSP proteins were concentrated using an Amicon centrifugal concentrator (10 kDa MWCO, Millipore) to a concentration of 4.5 mg/mL, measured by A_280_ using extinction coefficients of 26,930 and 36,900 M^−1^•cm^−1^ for MSP1E3D1 and MSP2N2, respectively. The protein was flash frozen on liquid nitrogen and stored at −80 °C.

### Methanotroph cell growth

*Mm. alcaliphilum* 20Z and *Mm. buryatense* 5GB1C were cultured following established methods^4,52^. Briefly, 12 L bioreactor cultures were grown in 1X modified nitrate mineral salts (NMSA) medium, 0.5 M NaCl (*Mm. alcaliphilum* 20Z) or 0.130 M NaCl (*Mm. buryatense* 5GB1C), 2.3 mM phosphate buffer, 50 mM carbonate buffer, pH 9.5, supplemented with 40 µM CuSO_4_•5H_2_O and trace elements solution (*Mm. alcaliphilum* 20Z 2000X stock solution: 0.5 g/L Na_2_•ETDA, 0.2 g/L FeSO_4_•7H_2_O, 0.01 g/L ZnSO_4_•7H_2_O, 0.003 g/L MnCl_2_•4H_2_O, 0.03 g/L H_3_BO_3_, 0.02 g/L CoCl_2_•6H_2_O, 0.002 g/L NiCl_2_•6H_2_O, 0.003 g/L Na_2_MoO_4_•2H_2_O; *Mm. buryatense* 5GB1C 500x stock solution: 1.0 g/L Na_2_•EDTA, 2.0 g/L FeSO_4_•7H_2_O, 0.8 g/L ZnSO_4_•7H_2_O, 0.03 g/L MnCl_2_•4H_2_O, 0.03 g/L H_3_BO_3_, 0.2 g/L CoCl_2_•6H_2_O, 0.02 g/L NiCl_2_•6H_2_O, 0.05 g/L Na_2_MoO_4_•2H_2_O). *Mc*. sp. str. Rockwell cells were cultured in 1X NMS medium, 3.9 mM phosphate buffer, pH 6.8, supplemented with 50 µM CuSO_4_•5H_2_O, 40 µM FeSO_4_•7H_2_O, and trace elements solution (500X stock solution: 0.288 g/L ZnSO_4_•7H_2_O, 0.166 g/L MnCl_2_•4H_2_O, 0.062 g/L H_3_BO_3_, 0.048 g/L Na_2_MoO_4_•2H_2_O, 0.048 g/L CoCl_2_•6H_2_O, 0.083 g/L KI)^3^. These cultures were grown under a continuous gas flow using a 1:3 methane-to-air ratio at 1.2 L/min at 30 °C with 300 rpm agitation. All bioreactor cultures were harvested at an OD_600_ of 8–10, centrifuged at 8000×*g* for 1 h, flash frozen in liquid nitrogen, and stored at −80 °C.

### Membrane isolation

Membranes from the three methanotrophs were isolated following established methods^3,4,52^. Ten grams of cells were resuspended in 100 mL of 25 mM PIPES, pH 7.3, 500 mM NaCl (*Mm. alcaliphilum* 20Z) or 250 mM NaCl (*Mm. buryatense* 5GB1C), supplemented with EDTA-free protease inhibitor tablets (Roche). Resuspended cells were sonicated for 1.5 min with an 1 s on, 3 s off interval at 25% sonication amplitude. *Mc*. sp. str. Rockwell cells (16 g) were resuspended in 70 mL of 25 mM PIPES, pH 7.3, 250 mM NaCl, supplemented with 500 µM CuSO_4_•5H_2_O, and sonicated for 7 min with an 1 s on, 3 s off interval at 25% sonication amplitude. All lysed cells were centrifuged at 8000×*g* for 1 h at 4 °C. The supernatant was then centrifuged at 100,000×*g* for 1 h at 4 °C. The resulting membrane pellet was washed twice in a Dounce homogenizer with 25 mM PIPES, pH 7.3., 250 mM NaCl. 1 mL aliquots of membranes (5–10 mg/mL) were flash frozen in liquid nitrogen and stored at −80 °C. Protein concentrations were measured using the DC Lowry Assay (Bio-Rad) with BSA as a standard.

### pMMO solubilization

pMMO was solubilized from the membranes using 1.2 mg of n-Dodecyl β-D-maltoside (DDM) (Anatrace) per 1 mg of protein at 4 °C for 1 h^3,4,52^. Membranes were pelleted at 100,000×*g* for 30 min at 4 °C, and the solubilized protein fraction was collected and buffer exchanged into 25 mM PIPES, pH 7.3, 250 mM NaCl, 0.02% DDM using a 100 kDa MWCO Amicon centrifugal concentrator (Millipore). Protein concentrations were measured using the DC Lowry Assay (Bio-Rad) with BSA as a standard.

### pMMO reconstitution into nanodiscs using dialysis

20Z- and Rockwell-pMMO were reconstituted into nanodiscs via dialysis using the membrane scaffold proteins MSP1E3D1 or MSP2N2 and 1-palmitoyl-2-oleoyl-sn-glycero-3-phosphocholine (POPC) lipids. A stock of 50 mM POPC was prepared from POPC powder (Avanti) in 100 mM sodium cholate, 25 mM PIPES, pH 7.3 and 250 mM NaCl. POPC was dissolved into the buffer by cycling the glass tube through 20 s of vortexing, a 20 s sonication step in an ultrasonic bath, and a 20 s incubation at 50 °C in a heat bath; this cycle was repeated until the lipids dissolved. For 20Z-pMMO, a pMMO trimer:MSP2N2:POPC molar ratio of 1:13:2340 was used for 5–10 mL reconstitutions in 20 mM PIPES, pH 7.3, 250 mM NaCl, 0.02% DDM buffer. The final concentration of the components in the reconstitution mixture was 3.33 μM pMMO trimer, 43.3 μM MSP2N2, 7.8 mM POPC, and 20 mM sodium cholate. For 20Z-pMMO using MSP1E3D1, the reconstitution molar ratio of pMMO:MSP1E3D1:POPC was 1:13:390 with final concentrations of 3.33 μM pMMO trimer, 43.3 μM MSP1E3D1, 1.3 mM POPC, and 20 mM sodium cholate. For Rockwell-pMMO using MSP1E3D1, the molar ratio of pMMO:MSP1E3D1:POPC was also 1:13:390. The reconstitution mixtures were rotated on a tube rotator at 4 °C for 1 h, followed by an overnight dialysis at 4 °C using a 10 kDa MWCO Slide-A-Lyzer dialysis cassette (ThermoFisher). For 20Z-pMMO, the reconstituted mixture was buffer exchanged into 20 mM PIPES, pH 7.3, 50 mM NaCl, and loaded onto a 5 mL or 10 mL HiTrap Q FF anion exchange chromatography column (GE Healthcare). A 50–800 mM NaCl gradient was used to separate empty nanodiscs and pMMO-embedded nanodiscs. Empty nanodiscs eluted at 300 mM NaCl, and 20Z-pMMO nanodiscs eluted at 600 mM NaCl. pMMO-nanodisc fractions were collected and concentrated using a 100 kDa MWCO Amicon centrifugal concentrator (Millipore). The 20Z-pMMO (post anion exchange) samples were loaded onto a Superose 6 Increase 10/300 GL column (GE Healthcare). Fractions corresponding to the pMMO-nanodisc complex were collected and concentrated using a using a 100 kDa MWCO Amicon centrifugal concentrator (Millipore). Protein concentrations of 20Z-pMMO nanodisc samples were measured using the DC Lowry assay with BSA as a standard. Copper content of 20Z-pMMO samples was determined using inductively coupled plasma optical emission spectrometry (ICP-OES), and copper content of Rockwell-pMMO samples was determined by ICP-MS, both at Northwestern University’s Quantitative Bio-element Imaging Center (QBIC). Copper concentrations were quantified using 0–500 ppb copper standards (Inorganic Ventures).

### pMMO reconstitution into nanodiscs using Bio-Beads

For copper supplementation experiments, Rockwell-pMMO was reconstituted into MSP1E3D1 nanodiscs using Bio-Beads (Bio-Rad). pMMO, MSP1E3D1, and POPC were mixed together for 2 h at 4 °C at a molar ratio of 1:4:240 pMMO:MSP1E3D1:POPC. For copper supplementation experiments, one molar equivalent of CuSO_4_·5H_2_O per solubilized Rockwell-pMMO protomer was added into the mixture. Self-assembly of the nanodisc was initiated by adding 0.5 g/mL wet Bio-Beads to the mixture followed by rotating on a tube rotator for 2 h at 4 °C. Wet Bio-Beads were prepared by mixing dry Bio-Beads with 25 mM PIPES, pH 7.3, 250 mM NaCl. Wet Bio-Beads were weighed by decanting onto a weigh boat followed by removal of the excess liquid with a Pasteur pipet. After reconstitution, Bio-Beads were removed from the nanodisc mixture by passing the mixture through a 0.22 µm syringe filter. The nanodiscs were then concentrated and purified by size-exclusion chromatography using a Superose 6 Increase 10/300 GL column (GE Healthcare). Fractions were collected and concentrated using a 100 kDa MWCO Amicon centrifugal concentrator (Millipore).

Empty nanodiscs could not be separated from the Rockwell-pMMO nanodisc samples, so the DC-Lowry Assay could not be used for accurate protein concentration measurements. Instead, Rockwell-pMMO nanodisc complexes were quantified using SDS-PAGE and ImageJ software^53^. Solubilized Rockwell-pMMO of known concentration (measured using the DC Lowry assay, Bio-Rad) was loaded onto a 15% SDS-PAGE gel at concentrations of 4, 2, and 1 mg/mL. ImageJ was then used to generate a standard curve, correlating the intensity of the PmoB subunit band with the known concentrations. The PmoB subunit was used for the standard curve since it was well separated from the PmoA, PmoC, and MSP bands on the gel. The concentration of the Rockwell-pMMO nanodisc sample was measured by comparing the intensity of its PmoB band against the standard curve generated above. Copper content was determined using inductively coupled plasma mass spectrometry (ICP-MS) at Northwestern University’s Quantitative Bio-element Imaging Center (QBIC). Copper concentrations were quantified using 0–500 ppb copper standards (Inorganic Ventures).

### Cryo-EM sample preparation and data acquisition

Freshly purified 20Z-pMMO sample in MSP2N2 nanodisc (3 µL at ~0.5 mg/mL) was deposited onto glow-discharged 400 mesh 1.2/1.3 C-Flat grids (Protochips). The Vitrobot Mark IV (FEI) sample chamber was kept at 100% relative humidity and the grid was blotted for 5–8 s before plunge freezing in a liquid ethane bath cooled by liquid nitrogen. The grids were imaged using a JEOL 3200FS microscope operating at 300 kV. Data were acquired on a K2 summit camera (Gatan) using Leginon^54^ with a defocus range between 1.5–3.5 µm using counting mode with a pixel size of 1.1 Å. Movies were recorded for 6 s exposure with a dose rate of approximately 8e-/pix/s (equivalent to 6.6e-/Å^2^/s on the plane of the sample).

Recorded movies were subjected to gain correction and then beam-induced motion correction with MotionCor2^55^. Following contrast transfer function (CTF) estimation with CTFFIND4^56^, micrographs with the best quality were then selected for further processing. Particles were then picked, extracted, and classified for 2D classification by applying C3 symmetry using the Scipion^57^ software environment using XMIPP programs^58,59^. Three best 2D classes representing different orientations of 20Z-pMMO in nanodiscs are shown in Fig. 4.

### ^13^C methane oxidation activity assay

Methane oxidation activity levels of pMMO-nanodisc complexes were performed^4^. Rockwell-pMMO in nanodiscs (~2–4 mg/mL) was resuspended in 25 mM PIPES, pH 7.2, 250 mM NaCl in 100 µL reactions containing reductant (excess duroquinol) in 2 mL screw top vials sealed with septa (Agilent). A 1 mL volume of headspace gas was withdrawn from the reaction vial and replaced with 1.5 mL of ^13^C methane gas (Sigma-Aldrich). All reactions were performed at 30 °C and 200 rpm for 5 min. The reactions were placed on ice for 5 min followed by quenching with 500 µL of chloroform containing 1 mM dichloromethane. The reaction was vortexed at 2000 rpm for 10 min and centrifuged at 2,000×*g* for 30 min. 2.5 µL of the chloroform mixture was injected into a PoraBOND Q column (25 m x 250 µm x 3 µm) on an Agilent 7890B/5977A MSD GC/MS instrument with a split ratio of 10:1. The column was under a constant flow of 1.2 mL/min of helium gas. The GC protocol was as follows: oven temperature was maintained at 80 °C for 3.5 min, ramped 50 °C/min to 150 °C and held for 1.5 min, and then ramped 15 °C/min to 300 °C and held for 1 min. The MS instrument protocol was as follows: 230 °C ion source temperature, 150 °C quad temperature, 70 eV, and a detector voltage of 2999 V. Ion masses 31, 33, and 49 were monitored for detection of ^12^C methanol, ^13^C methanol, and dichloromethane with dwell times of 10, 100, and 10 ms, respectively. ^13^C methanol concentrations were quantified using a standard calibration curve and the dichloromethane internal standard.

### Native mass spectrometry analysis

pMMO-nanodiscs samples for nTDMS analysis were dialyzed overnight using 10 kDa MWCO Slide-A-Lyzer MINI dialysis devices (Thermo Scientific) into 200 mM ammonium acetate, pH 7.2 (adjusted using ammonium hydroxide), and concentrated to approximately 30 μM pMMO-nanodisc complex. For detergent-solubilized pMMO samples, pMMO solubilized in DDM detergent were buffer exchanged into 200 mM ammonium acetate, pH 7.2, 0.155% (w/v) Triton X-100. Samples were analyzed using a Q Exactive HF mass spectrometer with Extended Mass Range and data were collected using XCalibur QualBrowser 4.0.27.10 (Thermo Fisher Scientific, Waltham, MA). The nTDMS platform employs direct infusion of sample into a native electrospray ionization (nESI) source held at +2 kV, C-trap entrance lens voltage setting between 1.8–4 V, HCD gas pressure setting between 2–4 V, and CID voltage set at 50–100 V for desalting and 150–195 V for protein ejection from detergent micelles or nanodisc complexes. The nTDMS platform is coupled to a three-tiered tandem MS process. The first step in the process^19^ is the analysis of the intact protein complex (MS^1^), which provides the total mass (reported as a deconvoluted neutral average mass value). In stage two, the complex is collisionally activated with nitrogen gas to eject monomers (MS^2^), thereby liberating the subunits that comprise each intact complex. In stage three, further vibrational activation of the ejected subunits via collisions with nitrogen gas yields backbone fragmentation products from each monomer (MS^3^) that are recorded at isotopic resolution (120,000 resolving power at *m/z* 400). These fragments are used to characterize the primary sequence of the monomers and localize posttranslational modifications. Intact mass values for pMMO complexes and ejected subunits, the MS^1^ and MS^2^ meassurements, were determined by deconvolution to convert data from the *m/z* to the mass domain using MagTran 1.03^60^ (mass range: 15,000–300,000 Da; max number of species: 10–15; S/N threshold: 1; mass accuracy: 0.05 Da; charge determined by: charge envelope only). Intact mass measurements are reported as neutral average masses; errors represent 1σ deviation from the mean of the masses calculated for of all sampled charge states. Fragmentation data were processed using mMass 5.5.0 (www.mmass.org), ProSight Lite 1.4^38^ (precursor mass type: average; fragmentation method: HCD; fragmentation tolerance: 25 ppm), and TDValidator 1.0^61^ (max ppm tolerance: 25 ppm; cluster tolerance: 0.35; charge range: 1–10; minimum score: 0.5; S/N cutoff: 3; Mercury7 limit: 0.0001; minimum size: 2) to assign recorded fragment ions to the primary sequence of the subunits. Specifically, ProSight Lite and TDValidator were used to analyze fragmentation spectra in medium throughput to assign and validate *b* and *y* fragment ions to the pMMO subunit sequences, and for generating a p-score. mMass was used to interrogate individual fragment ions within a spectrum, for example to examine metal binding and to validate that fragment ions correspond to their theoretical mass. The PmoA, PmoB, and PmoC subunits of 20Z-pMMO were identified by mapping backbone fragment ions to the amino acid sequence of pMMO subunits using ProSight Lite, with the p-scores^38^ of 3.2 × 10^–39^ (PmoA), 6 × 10^–15^ (PmoB), and 3.7 × 10^–14^ (PmoC). The PmoB and PmoC subunits of Rockwell-pMMO were identified using ProSight Lite with p-scores of 3.5 × 10^–8^ and 1.6 × 10^–34^, respectively^62^. Unexplained mass shifts (Δm) observed at the MS^1^, MS^2^, and MS^3^ levels for the intact complex and subunits, respectively, were manually interrogated using the UNIMOD database (http://www.unimod.org/modifications_list.php) as a reference for candidate modifications.

### Bottom-up proteomics methods

pMMO subunits from solubilized 20Z- and 5G-pMMO samples were separated using a reverse-phase HPLC 214TP54 analytical C4 column (Grace Vydac) on an Agilent 1100 HPLC^48^. Briefly, 100 µL of 30 µM pMMO trimer was injected onto the column and eluted using a gradient from 100% solvent A (63.75% formic acid, 10% acetonitrile, 5% *i*-PrOH) to 100% solvent B (70% formic acid, 30% *i*-PrOH). Eluted fractions were diluted 1:8 with water and digested with 2 μg pepsin (Promega) overnight at 37 °C. Pepsin was inactivated by heating samples at 95 °C for 10 min. The peptides were desalted on a C18 spin column (Pierce), and eluted with 80% acetonitrile in 0.1% formic acid. Samples were then lyophilized, resuspended with 5% acetonitrile in 0.1% formic acid, and injected onto a trap column (150 μm i.d. × 3 cm) coupled with a nanobore analytical column (75 μm i.d. × 15 cm, both ReproSil C18aq, 3 µm). Samples were separated using a linear gradient of solvent A (95% water, 5% acetonitrile, 0.1% formic acid) and solvent B (5% water, 95% acetonitrile, 0.1% formic acid). MS data were collected using a Velos Orbitrap Elite (Thermo) mass spectrometer operating in data-dependent top 10 mode. MS^1^ data were collected at a resolution of 60,000 at *m/z* 400 and an AGC target of 1,000,000. MS^2^ data were collected from the top 10 peaks in each precursor scan isolated with a 1.5 *m/z* isolation width fragmenting with CID with a normalized collision energy of 35 at an activation q of 0.25 and a duration of 10 ms at an AGC target of 10,000. Fragment ion spectra were recorded in the ion trap. The collected data were searched using Mascot 2.5 (Matrix Science) against custom proteomic databases for *Mm. buryatense* 5 G and *Mm. alcaliphilum* 20Z constructed from their published genomes^63,64^. Peptide MS^1^ tolerance was 15 ppm, while MS^2^ tolerance was 0.6 Da, and no cleavage enzyme was selected, so all subsequences for each protein in the database were queried. Variable modifications of deamidation of asparagine/glutamine and oxidation of methionine were allowed. Peptide fragmentation data were reported at 1% false discovery rate in Scaffold 4.5 (Proteome Software). Peptides containing post-translational modifications were validated by manual inspection of the tandem MS data. Bottom-up data proteomics have been deposited in the MASSive database with accession number MSV000083717 [10.25345/C5ND0J].

### Reporting summary

Further information on research design is available in the Nature Research Reporting Summary linked to this article.

## Supplementary information

Supplementary Information

Description of Additional Supplementary Files

Supplementary Data 1

Reporting Summary

Source Data

## Data Availability

Bottom-up proteomics data have been deposited in the MASSive database with accession number MSV000083717 [10.25345/C5ND0J]. Supplementary Data 1 contains expected and experimentally determined masses for nTDMS experiments. The source data underlying Fig. 7a, b and Supplementary Figs. 4 and 8 are provided as a Source Data file. A reporting summary for this Article is available as a Supplementary Information file. All other data supporting the findings of this study are available from the corresponding authors on reasonable request.
