# Supplementary material for: Native top-down mass spectrometry provides insights into the copper centers of membrane-bound methane monooxygenase
Source: Nat Commun. 2019 Jun 17;10:2675. doi: 10.1038/s41467-019-10590-6 (PMC6572826; doi:10.1038/s41467-019-10590-6)
Supplement: Supplementary file 1 — Supplementary Information [file 41467_2019_10590_MOESM1_ESM.pdf]

Native top-down mass spectrometry provides insights into the copper centers of membrane-bound methane monooxygenase

S. Y. Ro*, L. F. Schachner*, *et al.*

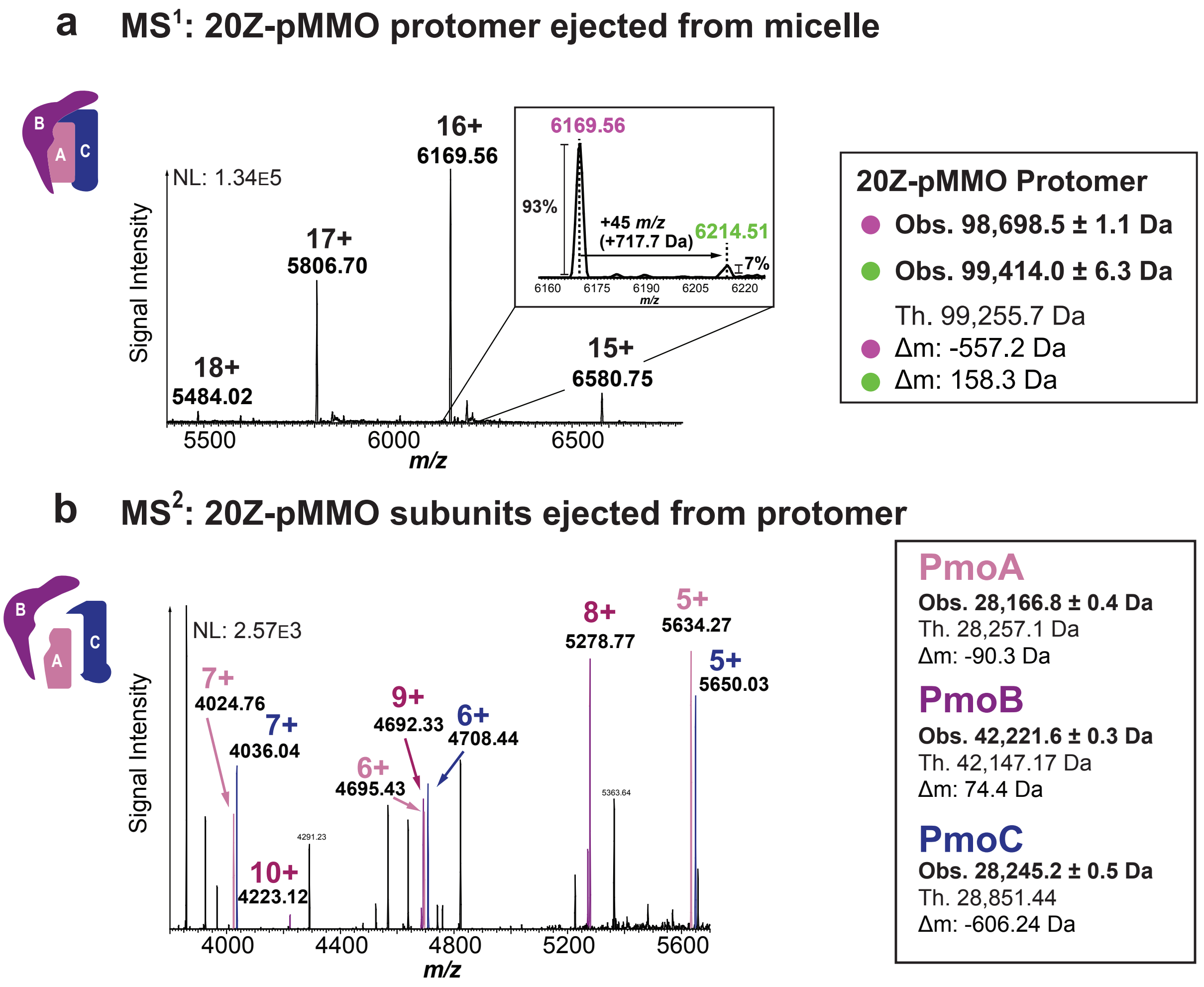

Supplementary Figure 1. nTDMS analysis of 20Z-pMMO in Triton X-100 micelles. a, Broadband MS^1 of 20Z-pMMO protomer upon ejection from a Triton X-100 micelle at CID of 195 V. The spectrum shows a charge state distribution of four protonated states of the pMMO protomer generated by native electrospray ionization (nESI). The inset is a zoom-in of the 16+ charge state, showing the presence of two species, one labeled in purple (93% of signal) and one in green (7% of signal). Charge state deconvolution of the two species yields a mass of 98,696 ± 1.1 Da (purple) and 99,414.0 ± 6.3 Da (green). The theoretical mass (abbreviated "Th." in figure) is derived from the unmodified subunits of pMMO and accounts for the cleavage of a known signal peptide in PmoB. **b**, MS^2 of 20Z-pMMO subunits ejected from the 16+ charge state of the protomer after activation by collisions with neutral gas at HCD of 150 V. The spectrum shows three protonated states for each of the three species detected, labeled pink, purple and blue for PmoA, PmoB, and PmoC, respectively. The addition of the measured masses of the ejected subunits yields 98,633.6 Da, which is 62.4 Da smaller than the major protomer mass measured in the MS^1. Unassigned peaks were attributed to Triton X-100 clusters that did not form discernible charge state distributions. NL values reflect maximum signal intensity in the spectrum.

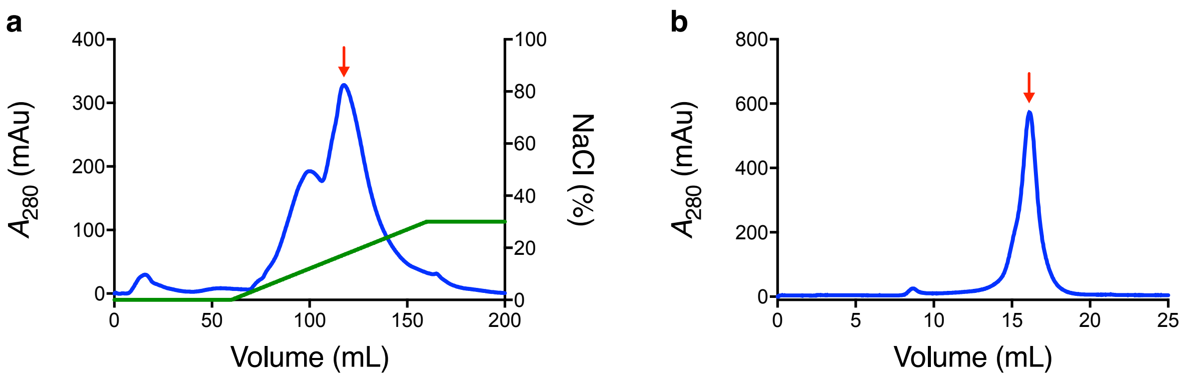

Supplementary Figure 2. Purification of 20Z-pMMO in MSP1E3D1 nanodiscs. a, HiTrap Q FF anion exchange chromatography purification showing 20Z-pMMO nanodisc complex (red arrow) followed by **b**, Superose 6 size exclusion chromatography purification. The absorbance at 280 nm (A_{280}) is shown in blue, and the % of 2 M NaCl in the gradient (v/v) is shown in green.

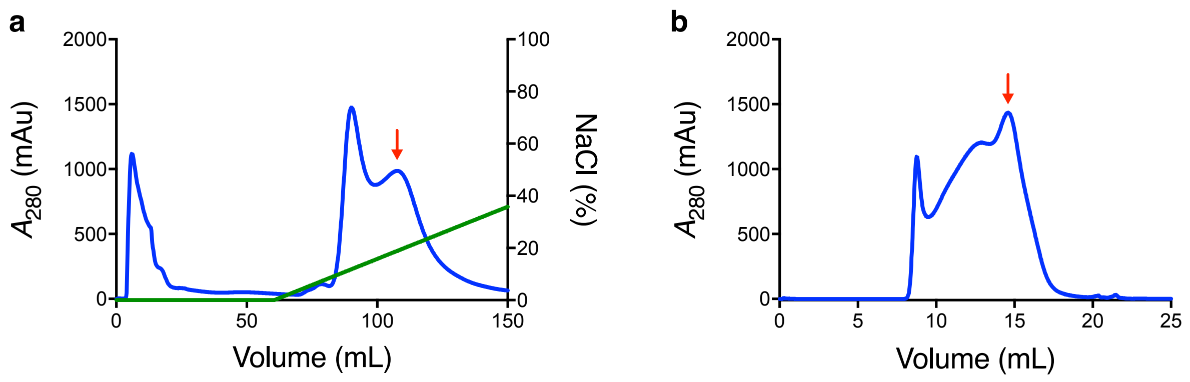

Supplementary Figure 3. Purification of 20Z-pMMO in MSP2N2 nanodiscs. a, HiTrap Q FF anion exchange chromatography purification showing 20Z-pMMO nanodisc complex (red arrow) followed by **b**, Superose 6 size exclusion chromatography purification. The absorbance at 280 nm (A_{280}) is shown in blue and the % of 2 M NaCl in the gradient (v/v) is shown in green.

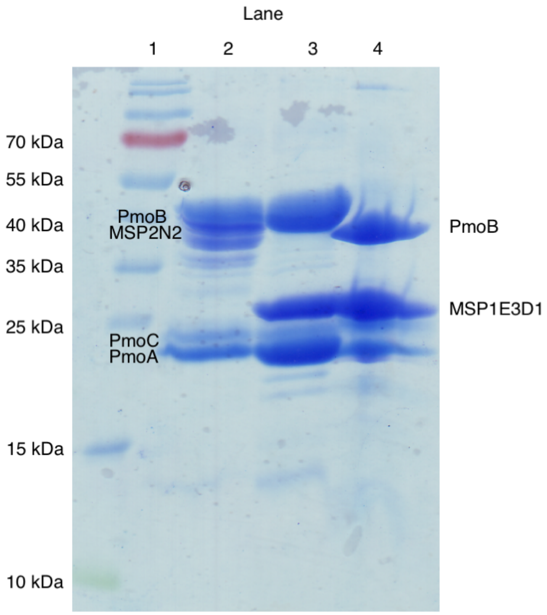

Supplementary Figure 4. SDS-PAGE gel of pMMO samples reconstituted in nanodiscs. Lane 1, MW markers; lane 2, 20Z-pMMO in MSP2N2 nanodiscs; lane 3, 20Z-pMMO in MSP1E3D1 nanodiscs; lane 4, Rockwell-pMMO in MSP1E3D1 nanodiscs. Source data are provided as a Source Data file.

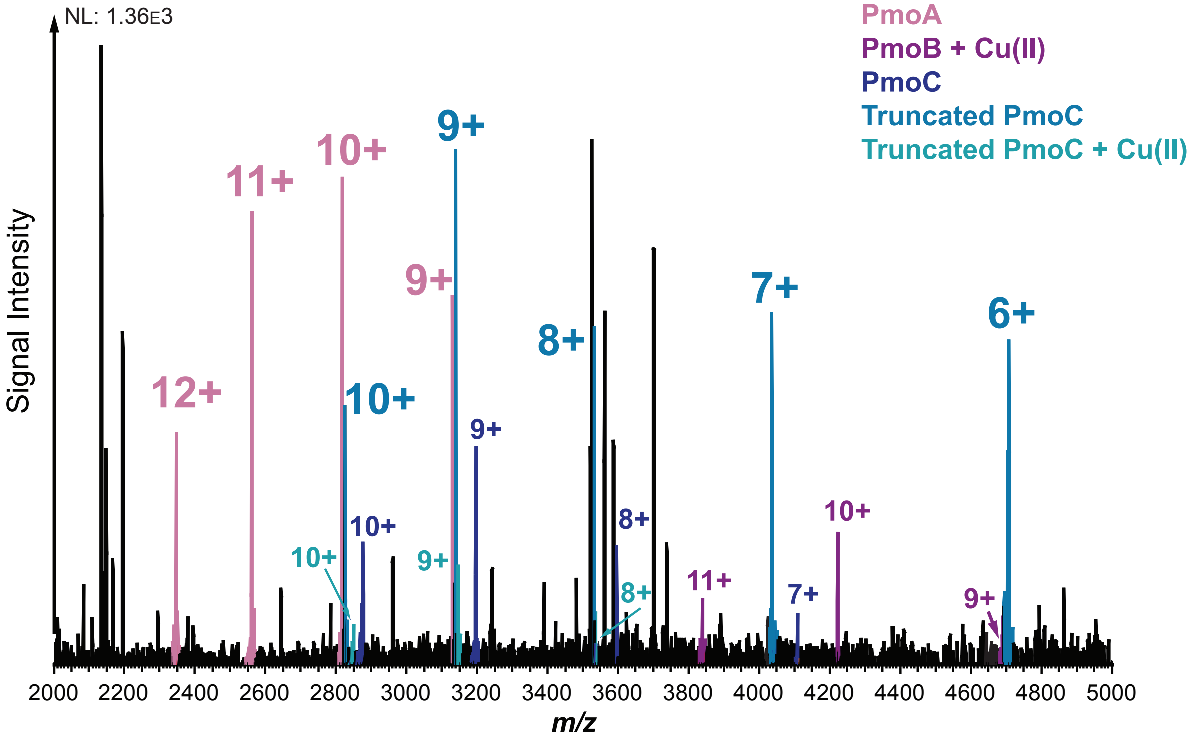

Supplementary Figure 5. Broadband MS^2 of 20Z-pMMO subunits ejected from MSP2N2 nanodiscs. Comparable results were obtained for 20Z-pMMO ejected from MSP1E3D1 nanodiscs. The spectrum shows charge state distributions for the five species detected, ejected from the nanodisc complex with CID of 195 V. The high intensity unidentified peaks can be attributed to charged lipid clusters of POPC used to assemble the nanodiscs. Notably, as indicated by the graphical fragment maps in Fig. 5b, PmoB includes residues His 33-Ile 414, consistent with a leader sequence that is cleaved post-translation. Moreover, PmoB was found to contain a methylation on Lys 36, which was fully localized by the tandem MS analysis of pepsin-digested peptides of 20Z-pMMO (Supplementary Figure 6a). PmoA was characterized to be Met$_{OFF}$ and NtAc. The MS^2 from the nanodisc reflects two populations of PmoC. One species was characterized to be Met$_{OFF}$ and NtAc. The major PmoC species is a truncated form lacking the first six N-terminal residues (MAATTE).

a **MS/MS of pepsin-digested peptide of 20Z-PmoB localizes methylation to Lys 36**

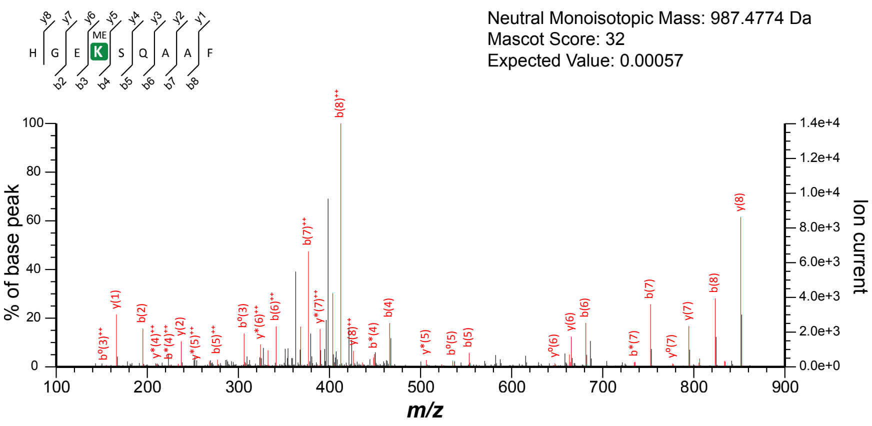

b **MS/MS of pepsin-digested peptides of 20Z-PmoC confirm N-terminal truncation**

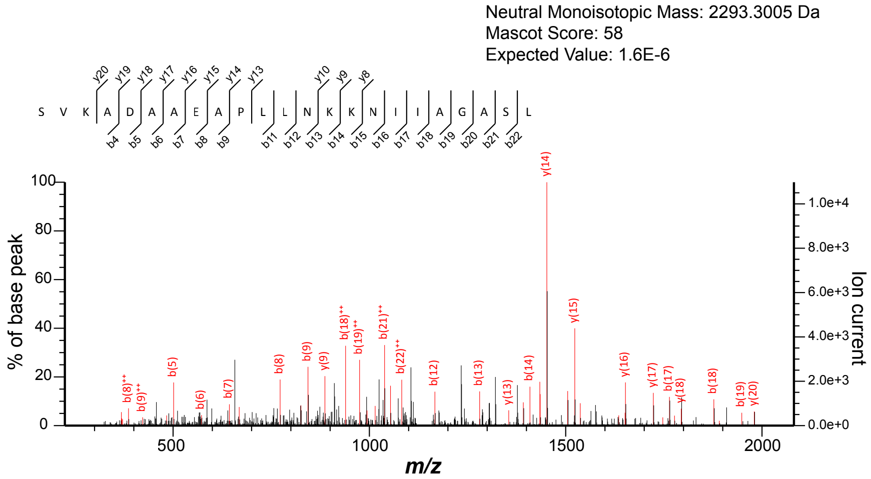

c **MS/MS of pepsin-digested peptides of 5G-PmoB localizes methylation to Lys 36**

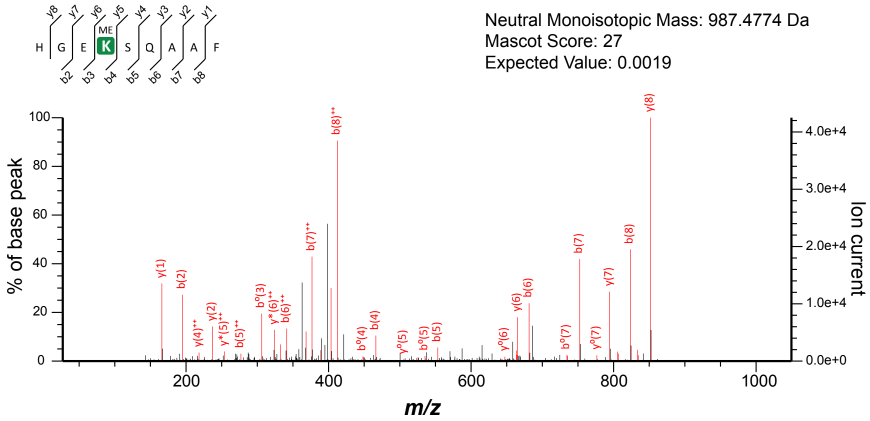

Supplementary Figure 6. **MS/MS fragmentation of pepsin-digested peptides to localize and confirm PTMs.** **a**, The detected 20Z-PmoB peptide contains a methylation on Lys 36. **b**, The detected 20Z-PmoC peptides indicate there is a population that contains N-terminal truncation of the first six residues MAATTE. **c**, The detected 5G-PmoB peptide contains a methylation on Lys 36. The y-axis on the left indicates the relative signal intensity as a percentage of the tallest peak in the spectrum; the y-axis on the right indicates the signal intensity in terms of ion current.

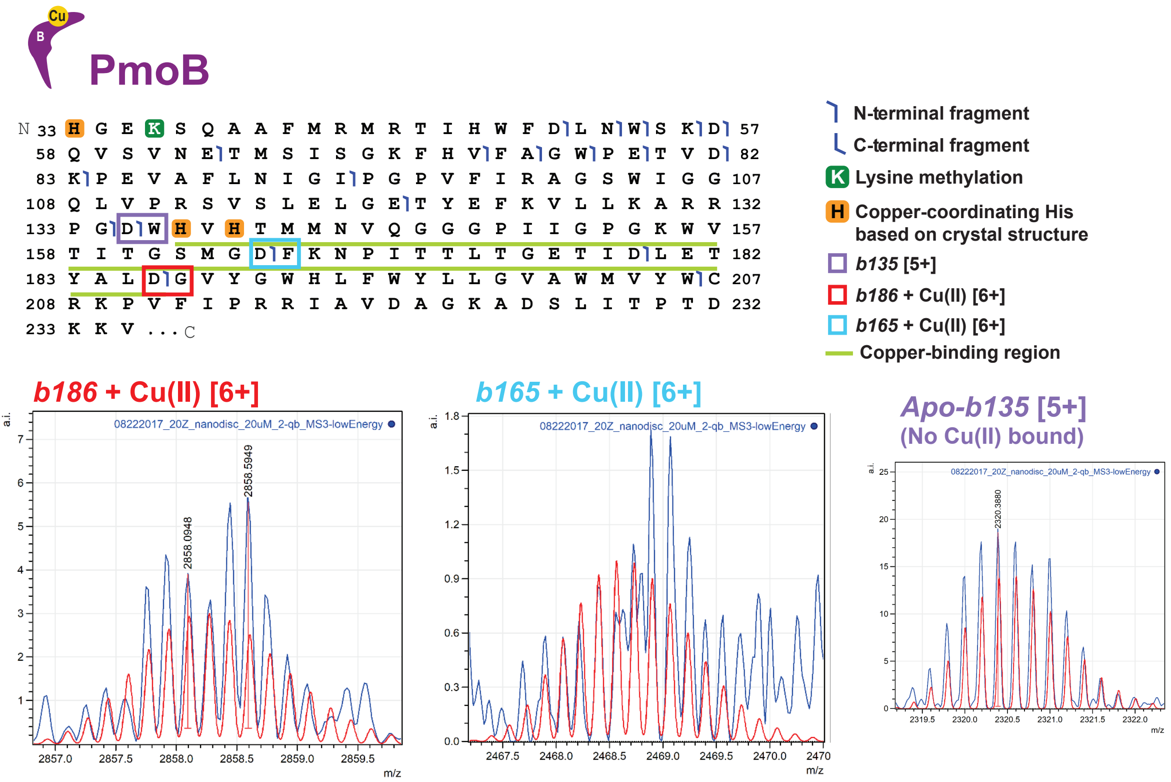

Supplementary Figure 7. **Pseudo-MS^3 fragmentation of 20Z-PmoB.** Fragmentation generates Cu(II)-bound *b* fragment ions (*b*$_{186}$, *b*$_{165}$) and an apo fragment ion (*b*$_{135}$) that help to verify the location of the copper center between residues Trp 136 and Asp 186.

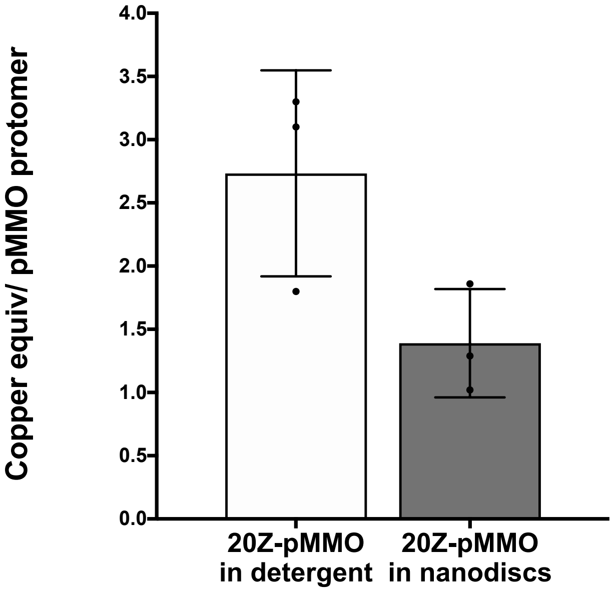

Supplementary Figure 8. Copper stoichiometry of 20Z-pMMO protomer in DDM and after MSP1E3D1 nanodisc reconstitution; n=3. The copper content was measured using ICP-OES and pMMO concentration was measured using the DC-Lowry assay. Error bars represent standard deviation represent individual measurements (black dots) of n=3. Source data are provided as a Source Data file.

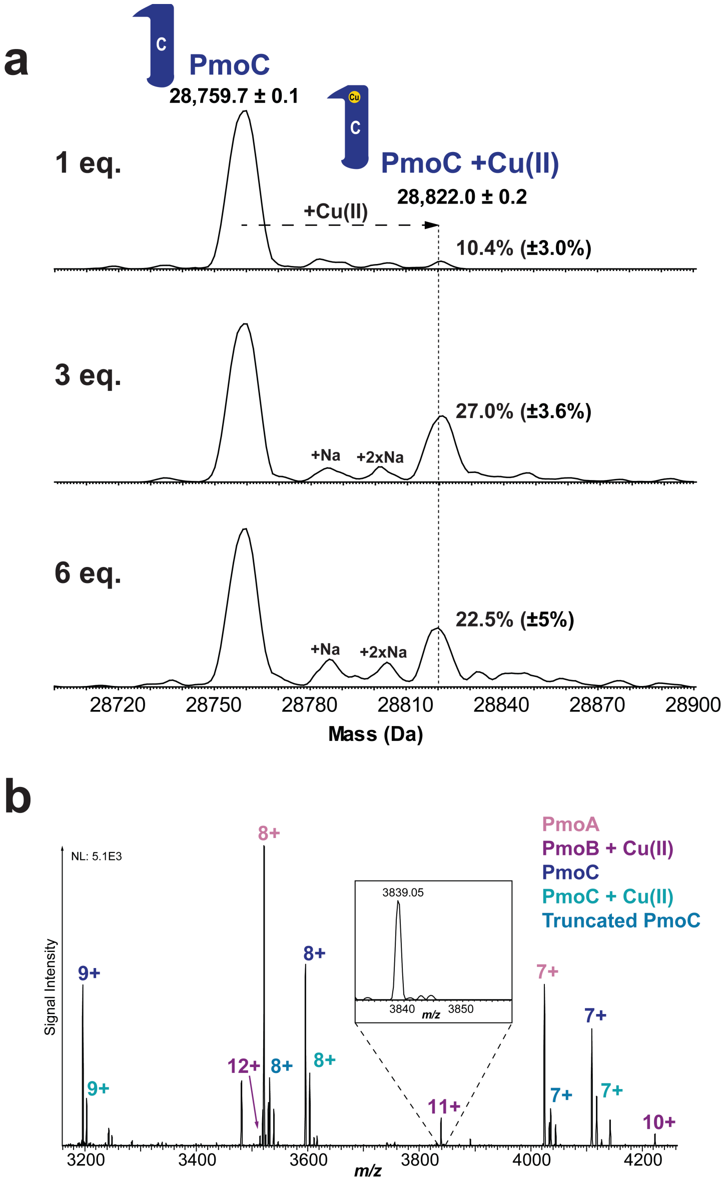

Supplementary Figure 9. nTDMS analysis of 20Z-pMMO in nanodiscs supplemented with exogenous copper ions post purification. a, Deconvoluted intact mass spectra reporting average masses and relative abundances of PmoC with and without Cu(II) ejected from 20Z-pMMO in MSP1E3D1 nanodiscs upon addition of 1, 3, and 6 molar equivalents (eq.) of copper per protomer to the electrospray buffer. **b,** Broadband mass spectrum of 20Z-pMMO subunits ejected from MSP1E3D1 nanodiscs incubated with 3 eq. of exogenous Cu(II) at CID energy of 195 V. The inset displays the 11+ charge state of copper-bound PmoB showing no additional copper binding after addition of 3 eq. of Cu(II)

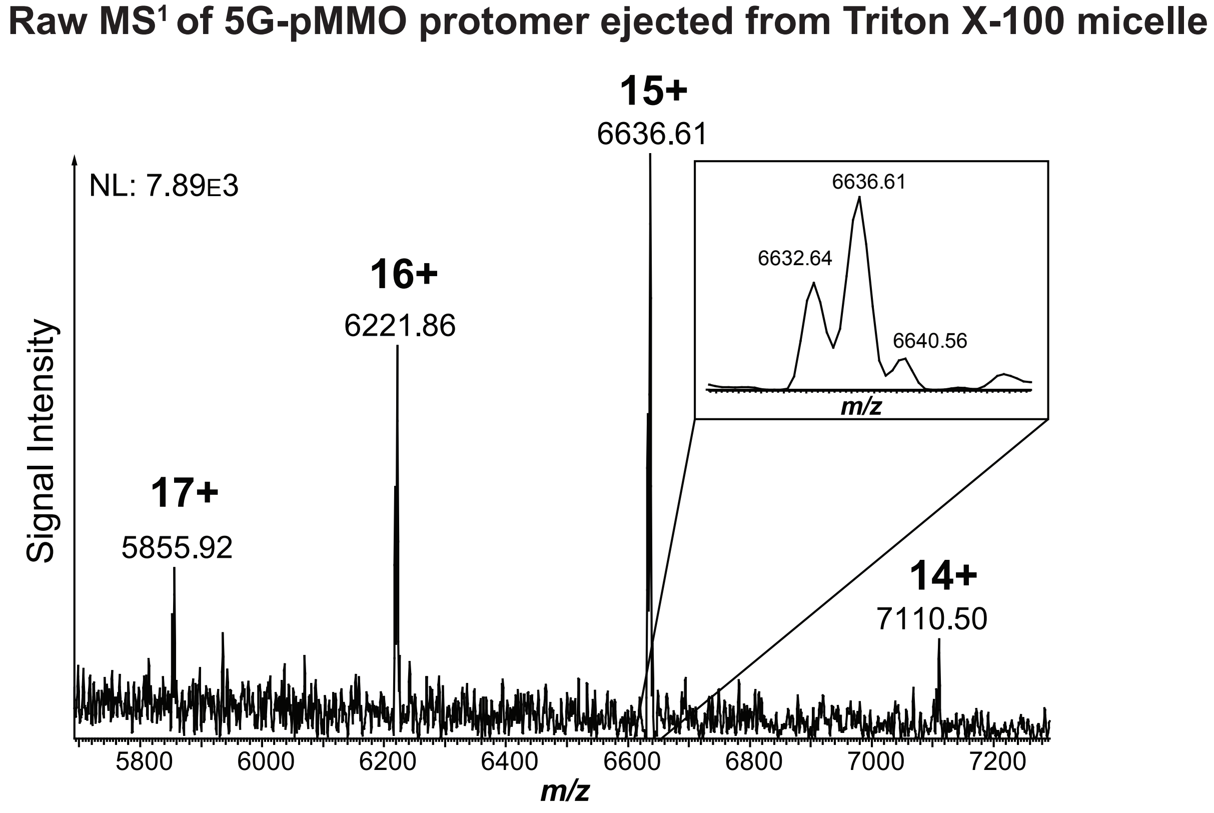

Supplementary Figure 10. Intact MS^1 spectrum of the 5G-pMMO protomer, showing a charge state distribution spanning the 14-17+ protonated states upon native ESI. The inset is a zoom-in of the 15+ charge state, showing the presence of three protomer species. See **Supplementary Figure 10a** for mass deconvolution of this spectrum.

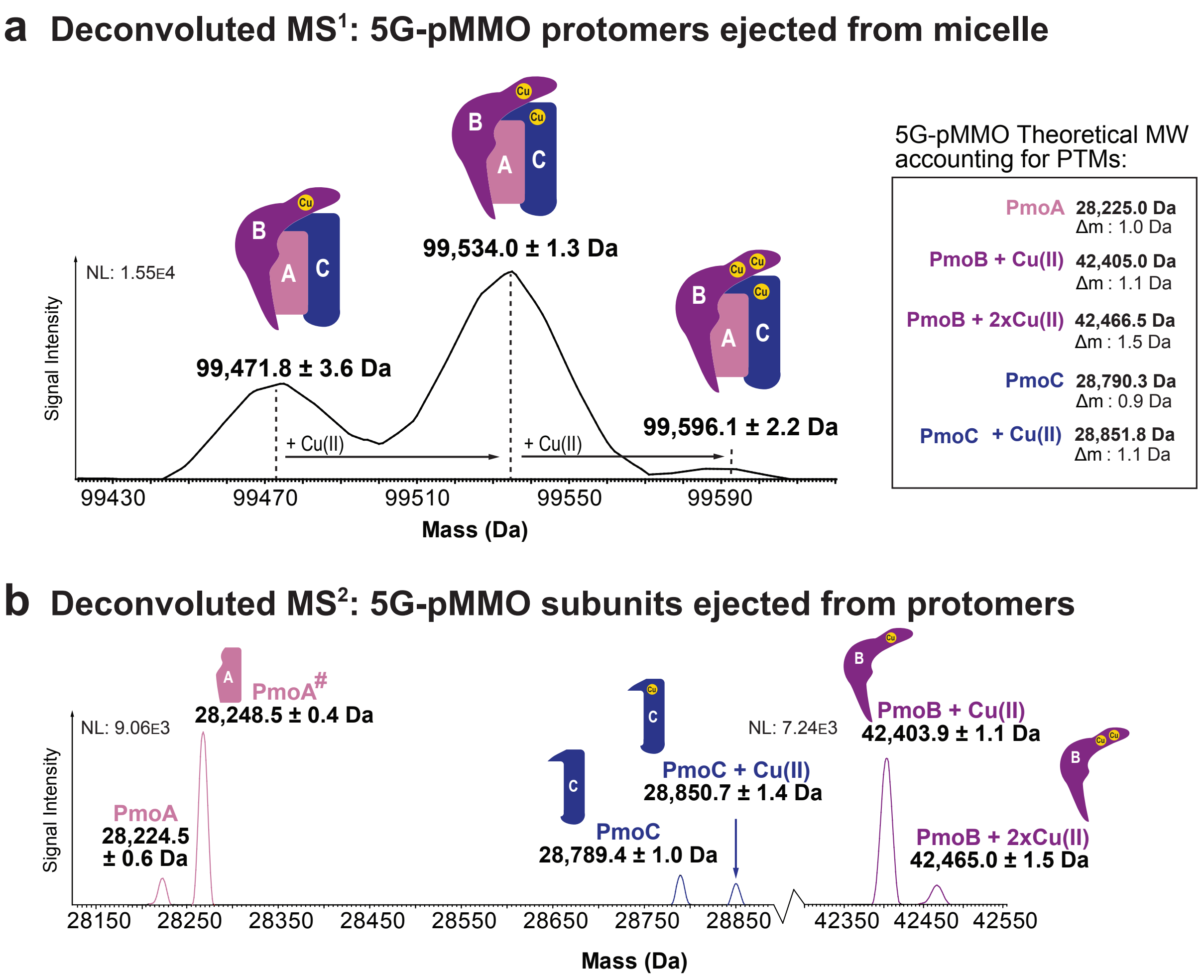

Supplementary Figure 11. nTDMS analysis of 5G-pMMO in Triton X-100 micelles. a, Deconvoluted MS^1 of 5G-pMMO protomer upon ejection from a Triton X-100 micelle with CID of 195 V. The deconvoluted spectrum highlights the three protomer species detected, which differ in mass by the mass of Cu(II). The panel on the right contains the theoretical molecular weights of the 5G-pMMO subunits, accounting for Met_{OFF} and NtAc of PmoA and PmoC and the methylation on PmoB Lys 36 characterized by the tandem MS of pepsin-digested peptides (**Supplementary Figure 6**). **b,** Deconvoluted MS^2 of 5G-pMMO subunits ejected upon protomer activation by collisions with neutral gas at the source. The spectrum shows detection of all subunits and indicates that PmoC and PmoB have mass shifts consistent with the binding of Cu(II). PmoB is found to mostly bind one Cu(II) ion; a small population of PmoB is shifted by a second mass of Cu(II), which could be binding at the bis-His site only observed in Bath-pMMO. PmoA is present as two populations: one with Met_{OFF} and NtAc and another with an additional uncharacterized 24.0 Da modification. The observed masses in the MS^2 were used to infer the identity of the protomers detected by MS^1 in **a**. The masses of the protomers measured in the MS^1 match most closely to PmoA with the 24.0 Da modification. NL values reflect maximum signal intensity in the spectrum.

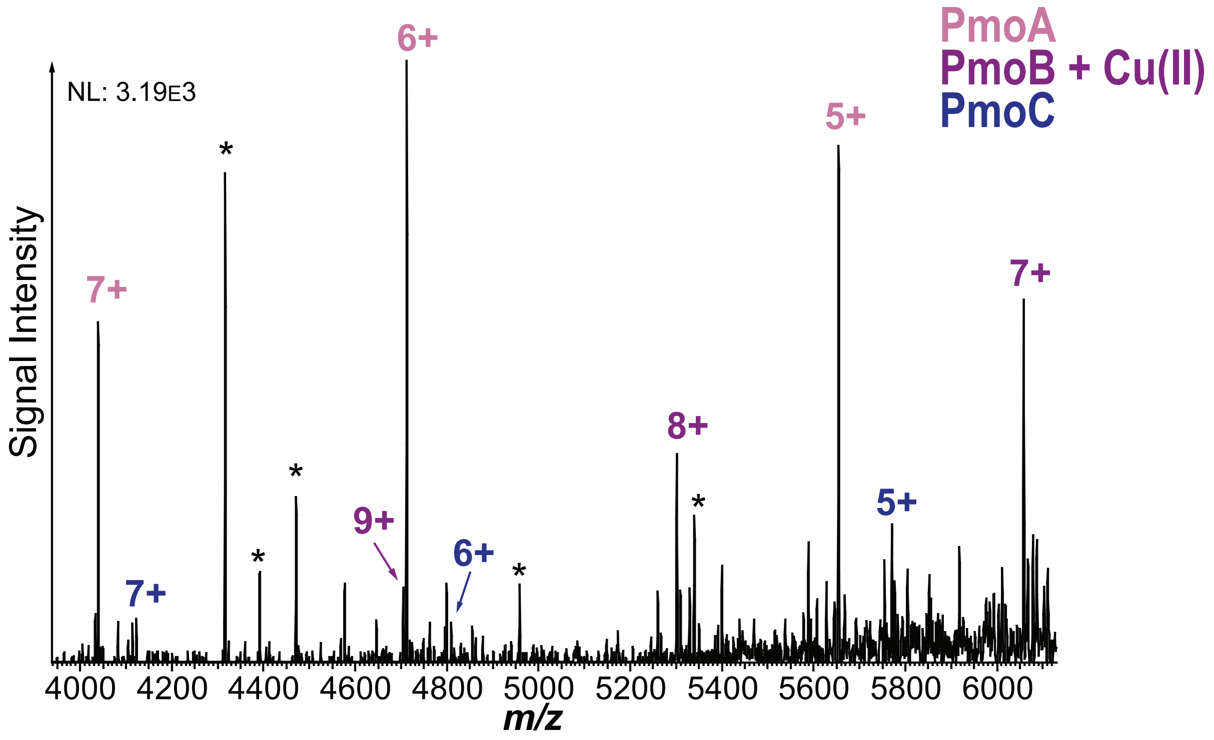

Supplementary Figure 12. Broadband MS^2 of 5G-pMMO subunits ejected from a Triton X-100 micelle using both CID and HCD activation (195 V and 150 V, respectively). The spectrum shows charge state distributions for PmoA, PmoB + Cu(II), and full-length PmoC. The peaks labeled with an asterisk (*)did not form discernible charge state distributions and may originate from Triton X-100 clusters. See **Supplementary Figure 10b** for mass deconvolution of this spectrum.

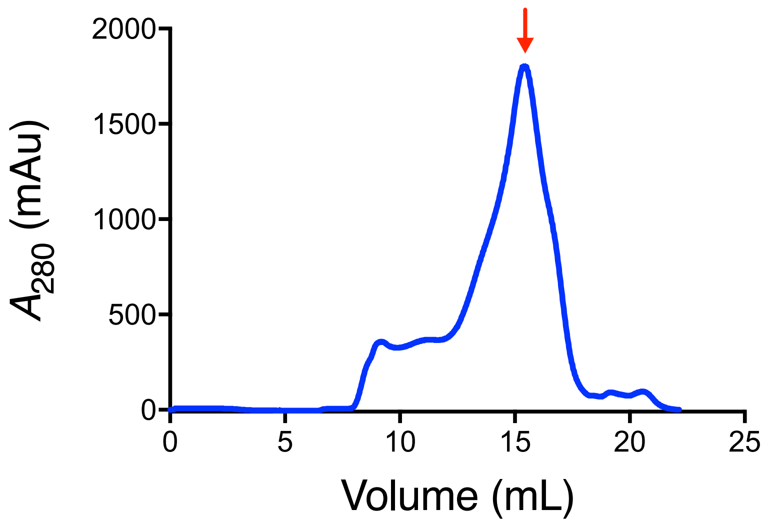

Supplementary Figure 13. Purification of Rockwell-pMMO in MSP1E3D1 nanodiscs. The Superose 6 size exclusion chromatography purification is shown with the Rockwell-pMMO nanodisc complex labeled (red arrow). The absorbance at 280 nm (A_{280}) is shown in blue.

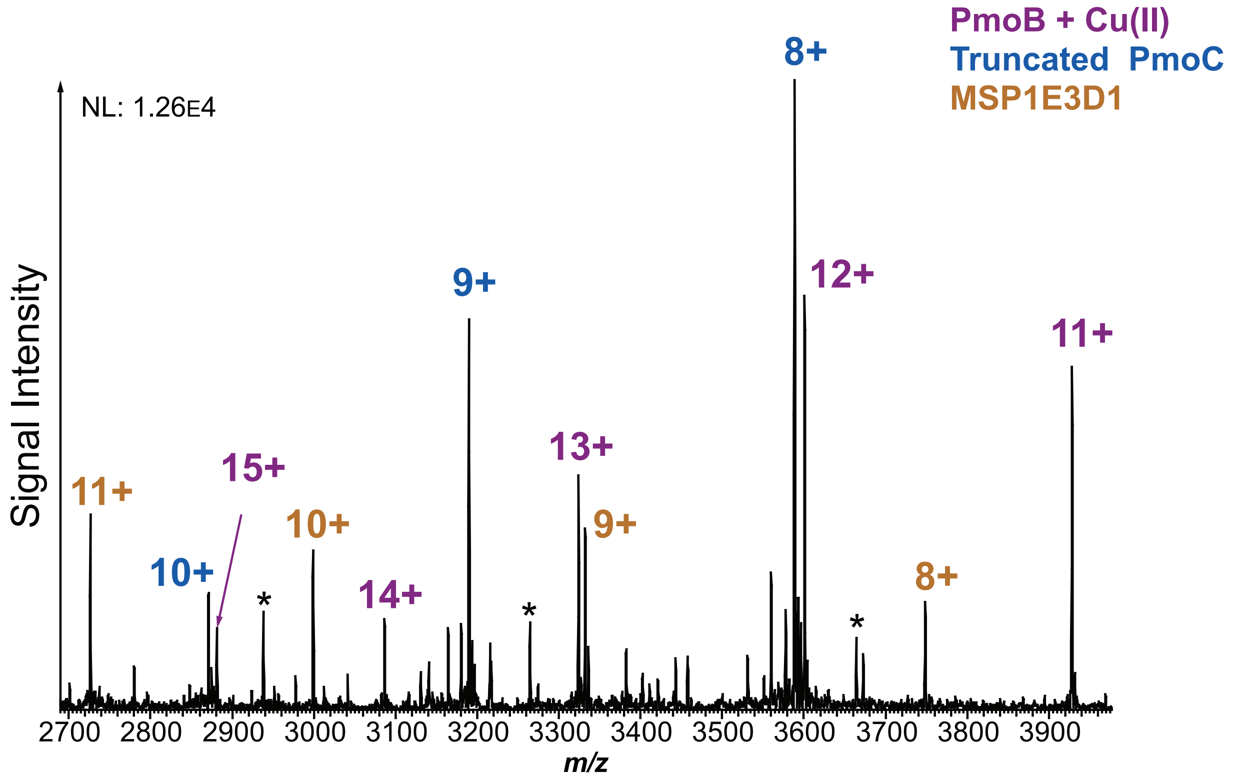

Supplementary Figure 14. Partial pseudo-MS^2 spectrum of Rockwell-pMMO subunits ejected from MSP1E3D1 nanodiscs. The spectrum shows charge state distributions for PmoB + Cu(II), truncated PmoC, and MSP1E3D1. The peaks identified with an asterisk (*) correspond to an uncharacterized protein impurity. Other unidentified peaks do not form discernible charge state distributions and likely result from lipid clusters of POPC released upon collisional activation of the nanodisc-pMMO complex.

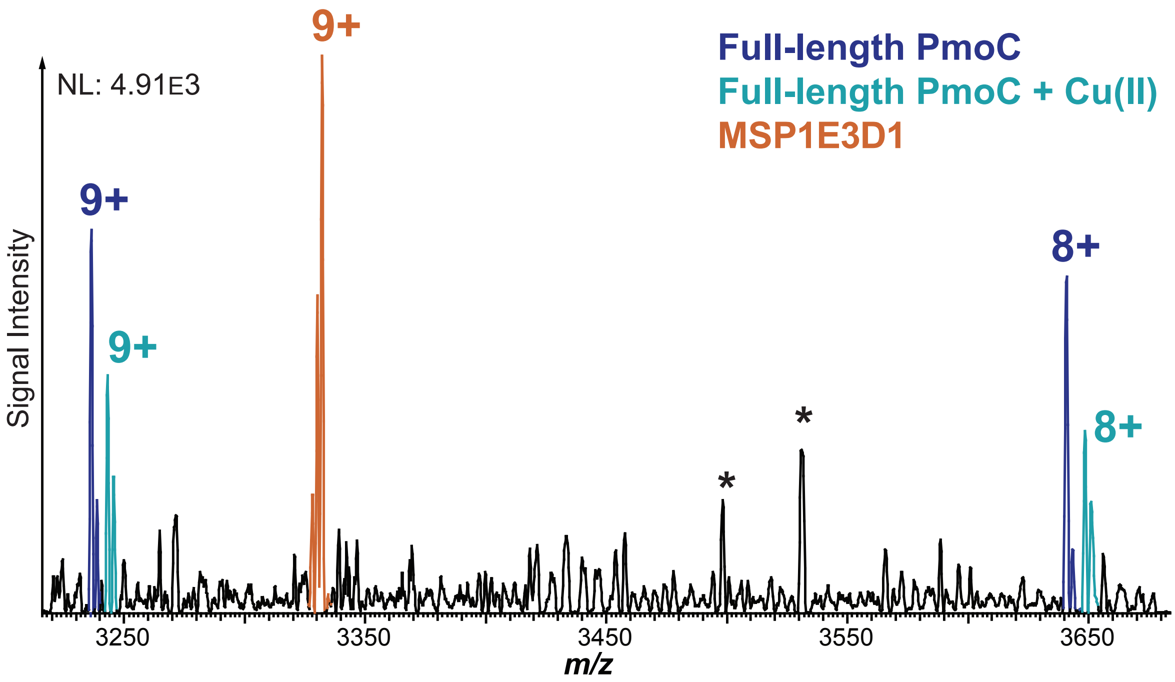

Supplementary Figure 15. Partial spectrum (~500 *m/z* wide) of the pseudo-MS^2 spectrum of Rockwell-pMMO subunits ejected from MSP1E3D1 nanodiscs supplemented with additional copper during reconstitution. The spectrum shows the charge states 8+ and 9+for full-length PmoC and full-length PmoC + Cu(II), and the 9+ charge state for the MSP1E3D1. The peaks identified with an asterisk (*) likely result from lipid clusters released upon collisional activation of the nanodisc-pMMO complex, as they correspond to known cluster masses or do not form discernible charge state distributions.
