# Supplementary material for: Native top-down mass spectrometry provides insights into the copper centers of membrane-bound methane monooxygenase
Source: Nat Commun. 2019 Jun 17;10:2675. doi: 10.1038/s41467-019-10590-6 (PMC6572826; doi:10.1038/s41467-019-10590-6)
Supplement: Supplementary file 2 — Description of Additional Supplementary Files [file 41467_2019_10590_MOESM2_ESM.docx]

**Description of Additional Supplementary Files**

**File Name: Supplementary Data 1**

**Description:** Theoretical and experimentally determined masses for nTDMS analysis, including lists of fragment ions used for 20Z-PmoA, 20Z-PmoB, 20Z-PmoC, Rockwell-PmoB, and Rockwell-PmoC proteoform identification and a table of theoretical and observed masses for all the pMMO proteoforms and complexes reported, including PTMs.
